# From Serum to Genome: γ-Glutamyltransferase Gene Family Variants Shape Ischemic Stroke Risk via Sex-Specific Gene–Environment Interactions

**DOI:** 10.3390/life16050721

**Published:** 2026-04-24

**Authors:** Maria Solodilova, Elena Drozdova, Iuliia Azarova, Marina Bykanova, Olga Bushueva, Anna Puchkova, Vyacheslav Puchkov, Maxim Freidin, Mikhail Churnosov, Alexey Polonikov

**Affiliations:** 1 Department of Biology, Medical Genetics and Ecology, Kursk State Medical University, 3 Karl Marx Street, Kursk 305041, Russia; solodilovama@kursksmu.net (M.S.); olga.bushueva@inbox.ru (O.B.); polonikovav@kursksmu.net (A.P.); vspuchkov21@gmail.com (V.P.); 2 Department of General Hygiene, Kursk State Medical University, 3 Karl Marx Street, Kursk 305041, Russia; drozdovael@kursksmu.net; 3 Department of Biological and Chemical Technology, Kursk State Medical University, 18 Yamskaya St., Kursk 305041, Russia; azzzzar@yandex.ru; 4 Laboratory of Biochemical Genetics and Metabolomics, Research Institute for Genetic and Molecular Epidemiology, Kursk State Medical University, 18 Yamskaya St., Kursk 305041, Russia; 5 Laboratory of Genomic Research, Research Institute for Genetic and Molecular Epidemiology, Kursk State Medical University, 18 Yamskaya St., Kursk 305041, Russia; marina.bickanova@yandex.ru; 6 Department of Twin Research and Genetic Epidemiology, King’s College London, Westminster Bridge Rd, London SE1 7EH, UK; maxim.freydin@kcl.ac.uk; 7 Department of Medical Biological Disciplines, Belgorod State University, 85 Pobedy Street, Belgorod 308015, Russia; churnosov@bsuedu.ru; 8 Laboratory of Statistical Genetics and Bioinformatics, Research Institute for Genetic and Molecular Epidemiology, Kursk State Medical University, 18 Yamskaya St., Kursk 305041, Russia

**Keywords:** ischemic stroke, gamma-glutamyl transferase, single nucleotide polymorphism, lifestyle factors, gene–gene interactions, gene–environment interactions

## Abstract

Serum gamma-glutamyltransferase (GGT) is a biomarker for cardiovascular disease, but the role of its encoding gene family in ischemic stroke (IS) is unknown. This pilot study of 1288 individuals (600 cases and 688 controls) investigated *GGT1*, *GGT5*, *GGT6*, and *GGT7* polymorphisms using the MassARRAY-4 system. Conventional single-variant, haplotype, and diplotype analyses were complemented by Model-Based Multifactor Dimensionality Reduction (MB-MDR) with stability assessment and model prioritization. Conventional analysis identified female-specific associations for three *GGT5* variants (rs8140505, rs2275984, and rs2267073; P_perm_ < 0.05). A common *GGT5* haplotype was protective in females (P_perm_ = 0.02). Diplotype analysis revealed joint effects of GGT genotypes on IS risk in females (FDR < 0.05). MB-MDR uncovered complex higher-order interactions (P_perm_ < 0.0001): in women, 12 models represented second-order interactions between smoking and individual GGT variants. In men, 8 models centered on *GGT1* rs5751909 spanning second- to fourth-order interactions with alcohol, smoking, and other GGT family members. All prioritized models passed FDR correction (q < 0.05) and achieved higher weighted composite scores. eQTL data linked these variants to regulatory networks controlling glutathione metabolism, oxidative stress, and inflammation. This study supports a novel hypothesis on the combined involvement of GGT gene family polymorphisms and pro-oxidant environmental factors in ischemic stroke predisposition, demonstrating that disease risk is shaped by sex-specific gene–environment interactions. The pronounced sexual dimorphism highlights the need for sex-specific personalized approaches: smoking cessation may be particularly impactful in women carrying *GGT5* risk variants, while alcohol moderation could be prioritized in men with *GGT1* risk variants.

## 1. Introduction

Stroke is the fourth leading cause of long-term disability and death worldwide, with a 50% increase in lifetime risk over the past 17 years [1,2]. Ischemic stroke (IS), the most common type, is caused by thrombotic or embolic events that impair cerebral blood flow [3]. Despite advances in acute treatment, mortality and disability from IS remain relatively high, reflecting its nature as a multifactorial disease resulting from a complex interplay of genetic, epigenetic, and environmental factors that influence the entire pathological continuum—from the development of atherosclerosis to brain injury and subsequent recovery [4,5,6,7]. A growing body of research identifies oxidative stress as a key pathological factor in cerebrovascular diseases, where an imbalance of reactive oxygen species overwhelms the body’s antioxidant defense mechanisms [8,9]. Among the various biomarkers of ischemic stroke associated with inflammation, endothelial dysfunction, and thrombosis, oxidative stress markers are of particular value, as they directly reflect the sustained imbalance between free radical generation and antioxidant defense capacity [8,9,10,11].

Within this framework, gamma-glutamyl transferase (GGT)—a membrane-bound enzyme that catalyzes the cleavage of the gamma-glutamyl bond in glutathione, a major antioxidant protecting cells against oxidative stress—has emerged as a biomarker of considerable interest. The GGT reaction is a crucial step in the γ-glutamyl cycle, facilitating the transport of amino acids across cell membranes and the recycling of glutathione [12,13]. Elevated serum GGT activity has been consistently associated with both increased risk and greater severity of ischemic stroke, independent of age, sex, and conventional risk factors [14,15,16,17,18,19,20,21,22,23,24]. Furthermore, Mendelian randomization studies have substantiated a causal relationship between circulating GGT levels and ischemic stroke susceptibility [25,26,27].

Despite the well-established association between elevated blood GGT levels and ischemic stroke risk—underscoring the role of redox imbalance in its etiology—the causal molecular mechanisms linking these phenotypes remain unresolved. GGT is thought to contribute to ischemic stroke pathogenesis through pro-oxidant effects, glutathione depletion, and amplification of inflammation, endothelial dysfunction, atherosclerosis, and oxidative damage [8,9]. However, it remains unclear whether elevated GGT-driven glutathione catabolism is an initial trigger of oxidative stress or a compensatory response to primary intracellular glutathione deficiency. We hypothesize that, due to its membrane-bound localization and central role in glutathione metabolism, GGT acts not merely as a biomarker but also as an integrative hub connecting intra- and extracellular redox homeostasis via interorgan glutathione exchange (liver to periphery) and precursor amino acid supply for de novo glutathione synthesis.

The GGT family includes at least eight putative proteins, namely GGT1, GGT2, GGT3P, GGT4P, GGT5, GGT6, GGT7, and GGT8P [28], but only GGT1 and GGT5 show catalytic activity [29]. Despite structural similarities, individual GGT enzymes may serve distinct tissue-specific roles. GGT1 is the primary source of serum gamma-glutamyl transferase activity [28]. Beyond glutathione catabolism, GGT1 and GGT5 also mediate inflammatory signaling by converting leukotriene C4 to leukotriene D4 [30,31]—lipid mediators involved in vascular tone, permeability, platelet function, and CNS neuromodulation. Thus, gamma-glutamyl transferases are not merely metabolic enzymes but also multifunctional candidates linking redox balance, inflammation, and cerebrovascular pathology.

Although polymorphisms in glutathione-related redox genes are well-known risk factors for ischemic stroke [32,33,34,35,36,37], the role of gamma-glutamyl transferase genes—despite GGT’s established status as a cardiovascular marker—remains unexamined in this context. Our team has pioneered research on GGT gene polymorphisms in stroke susceptibility [38,39]. This pilot study aims to: (1) investigate the involvement of *GGT5* and *GGT6* polymorphisms in ischemic stroke development; and (2) comprehensively assess interactions among *GGT1*, *GGT5*, *GGT6*, and *GGT7* variants, as well as their synergistic effects with smoking and alcohol consumption, on cerebrovascular disease risk.

## 2. Materials and Methods

### 2.1. Study Patients

#### 2.1.1. Study Population and Ethical Approval

Prior to enrollment in the study, all participants provided written informed consent. The study protocol was approved by the Ethical Review Committee of Kursk State Medical University (Approval No. 6, dated 14 May 2018). A cohort of 1288 unrelated individuals of East Slavic descent (Russians, Ukrainians and Belarusians) was enrolled in this study in accordance with the criteria and characteristics outlined in previous publications [33,35]. Ethnicity was determined based on self-identification and verified by the ethnicity of parents and grandparents. Specifically, individuals were classified as Russian only if both parents and all four grandparents were Russian. Participants with any non–East Slavic ancestry were excluded from the study.

#### 2.1.2. Study Participants and Clinical Characteristics

**Figure 1:**
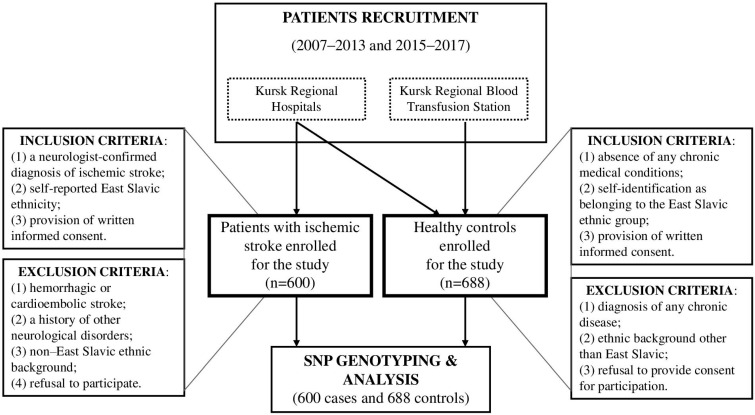
Flowchart diagram illustrating the inclusion and exclusion criteria for patient recruitment.

The study involved 600 patients with ischemic stroke, including 330 males and 270 females, with a mean age of 61.09 ± 9.77 years. All patients were hospitalized in the Neurological Division of the Regional Vascular Center, which is part of the Kursk Regional Clinical Hospital. Clinical examination, computed tomography, and/or magnetic resonance imaging of the brain were performed to confirm the diagnosis of ischemic stroke. Information regarding specific stroke subtypes—including large artery disease, small vessel disease, and cardioembolic stroke—was not available for the patients included in this study. The primary patient cohort was established through the consecutive screening of all hospitalized individuals. Figure 1 presents the flowchart diagram illustrating the inclusion and exclusion criteria for patient recruitment. Patients were enrolled in the ischemic stroke group if they met the following criteria: (1) a neurologist-confirmed diagnosis of ischemic stroke; (2) self-reported East Slavic ethnicity (Russian, Ukrainian or Belarusian); and (3) provision of written informed consent. Exclusion criteria included: (1) hemorrhagic or cardioembolic stroke; (2) a history of other neurological disorders; (3) non–East Slavic ethnic background; and (4) refusal to participate. The control group consisted of 688 healthy individuals without any chronic diseases, including 366 males and 322 females, with a mean age of 60.84 ± 7.45 years. The control group, recruited in our previous studies as described elsewhere [34,35,36,40,41], was fully matched to the ischemic stroke patient group by recruitment site, ethnicity, sex, and age. The inclusion criteria for the control group were as follows: (1) absence of any chronic medical conditions; (2) self-identification as belonging to the East Slavic ethnic group; and (3) provision of written informed consent. The exclusion criteria included: (1) diagnosis of any chronic disease; (2) ethnic background other than East Slavic; and (3) refusal to provide consent for participation.

#### 2.1.3. Assessment of Smoking and Alcohol Consumption

A validated brief screener [42] was used to assess environmental risk factors. Data on cigarette smoking use and alcohol consumption (beer, wine, and spirits) were collected through an interviewer-administered questionnaire. The frequency of alcohol intake was assessed by asking participants: “Do you consume any alcoholic beverages? If so, how many days per month or week?” Responses were then categorized as follows: never or once per month; 1–2 days per month; 1–2 days per week; 3–4 days per week; and five or more days per week. Based on these reported frequencies, participants were divided into two groups: (1) infrequent drinkers, defined as those consuming alcohol one to two days per month or less, and (2) regular drinkers, defined as those consuming alcohol at least one day per week. For the purposes of this analysis, the latter group was operationally designated as exhibiting a pattern of regular alcohol use. Smoking status was recorded dichotomously as ever-smoker versus never-smoker. Measures of smoking intensity, such as pack-years, were not assessed in this study. Baseline clinical and laboratory characteristics, along with data on environmental risk factors of the study patients, were described in detail in recent papers [38,39].

### 2.2. Genetic Analysis

#### 2.2.1. SNP Selection

The selection of *GGT5* and *GGT6* gene polymorphisms was conducted using the Candidate Gene SNP Selection (GenePipe) tool, available online at the SNPinfo Web Server (https://snpinfo.niehs.nih.gov/snpinfo/selegene.html, accessed on 15 May 2023). Common functional SNPs in the *GGT5* gene (rs2267073, rs2275984, and rs8140505) and the *GGT6* gene (rs11657054 and rs2100986) were selected for this study based on the following criteria: tagSNP rI ≥ 0.8, minor allele frequency (MAF) > 5%, and the presence of an eQTL associated with the expression levels of the corresponding genes in whole blood (data from the eQTLGen Consortium, https://www.eqtlgen.org/phase1.html, accessed on 15 May 2023) and other tissues (data from the GTEx project, https://www.gtexportal.org/home/, accessed on 15 May 2023). Polymorphisms of the *GGT1* and *GGT7* genes were selected and genotyped as described in our previous studies [38,39]. These data, along with genotype data for *GGT5* and *GGT6*, were used to conduct gene–gene and gene–environment interaction analyses.

#### 2.2.2. SNP Genotyping and Quality Control

Genetic analyses were conducted at the Research Institute for Genetic and Molecular Epidemiology, Kursk State Medical University, Kursk, Russia. Total DNA was extracted from the whole blood samples of the study patients using a two-step procedure involving phenol-chloroform extraction followed by ethanol precipitation. Genotyping was performed using iPLEX technology on the MassARRAY 4 system (Agena Bioscience, San Diego, CA, USA). The MassARRAY Assay Design Suite (https://agenacx.com, accessed on 15 May 2023) was used to select primer sets and design a multiplex panel for SNP genotyping. Primer sequences are available upon request. Five percent of the DNA samples were genotyped in duplicate, blinded to case–control status, to ensure quality control. Over 99% of the data were concordant.

A stringent quality control threshold was applied: any individual with missing genotype data for at least one of the nine SNPs was excluded from the study. Thus, all 1288 participants included in the final analyses had complete genotype calls for all nine polymorphisms, resulting in 100% call rates for both SNPs and samples. Initially, 11 SNPs were genotyped (Supplementary Table S1). Two SNPs (GGT7 rs6119534 and GGT1 rs5760492) showed significant deviation from Hardy–Weinberg equilibrium in the control group (*p* < 0.0001) and were therefore excluded from all subsequent analyses. The remaining nine SNPs, all of which conformed to HWE, were retained for the final analyses (Supplementary Table S1).

### 2.3. Statistical and Bioinformatics Analysis

#### 2.3.1. Power Calculations

Statistical power was calculated using the method described by Gauderman [43] for case–control studies. For single-variant associations, we evaluated three polymorphisms within *GGT5* (rs8140505, MAF = 0.27; rs2275984, MAF = 0.30; rs2267073, MAF = 0.44) and two within *GGT6* (rs11657054, MAF = 0.12; rs2100986, MAF = 0.15). Minor allele frequencies were derived from observed data and assumed to be similar across sexes. Assuming a multiplicative genetic model and a disease prevalence of 0.01, we estimated power to detect odds ratios (ORs) of 1.5, 2.0, and 2.5. Calculations were performed for the overall sample and separately for women and men. Multiple testing correction was applied using the Bonferroni method for five independent tests (α_corrected_ = 0.01). For the total sample, power exceeded 99% for all five SNPs at OR = 1.5 and reached 100% for OR ≥ 2.0. In sex-stratified analyses, power remained ≥99.8% for all SNPs at OR = 2.0. For *GGT6* variants power was 99.8% in men and 100% in women at OR = 2.0. These results confirm that the study was adequately powered to detect single-variant associations of moderate effect size in both the total sample and sex-stratified analyses. For gene–environment interactions, power was calculated for second-order interactions assuming an OR of 2.0, MAFs ranging from 0.15 to 0.45, and environmental factor prevalences of 0.38 (smoking) and 0.17 (alcohol). After Bonferroni correction for multiple tests (α_corrected_ = 6.5 × 10^−4^), estimated power was 90.1% under average conditions (MAF = 0.23, pE = 0.27).

#### 2.3.2. Single-Variant, Haplotype, and Replication Analyses

The first stage of the study involved analyzing allele and genotype frequencies of the *GGT5* and *GGT6* gene polymorphisms and assessing their associations with the risk of ischemic stroke. Allele and genotype frequencies in both the case and control groups were calculated and compared using Fisher’s exact test to detect significant deviations from Hardy–Weinberg equilibrium. The associations between *GGT5* and *GGT6* alleles and genotypes and the risk of ischemic stroke were quantified using odds ratios with 95% confidence intervals (95% CIs). Allele, genotype, and haplotype frequencies within the study cohorts, along with their correlations with disease susceptibility, were analyzed using PLINK software, version 1.9 [44]. The *p*-values (P_perm_) for the associations between alleles/genotypes and haplotypes with disease risk were calculated using adaptive permutation testing with the software tools PLINK and Haploview (version 4.2), respectively.

Association analyses were performed separately for the overall group and sex-stratified groups. Formal testing of predictor-by-sex interaction was performed using logistic regression with an interaction term (Predictor × SEX) for all nine SNPs (additive, dominant, and recessive models) and two environmental factors (smoking status, regular alcohol drinking), adjusted for age. Multiple testing was controlled using FDR with q < 0.05.

To replicate the SNP–disease associations identified in our study population, replication analyses were performed using independent European cohorts with genomic data available from the Cerebrovascular Disease Knowledge Portal (https://cd.hugeamp.org, accessed on 12 September 2025).

#### 2.3.3. Diplotype Associations of GGT Gene Family Polymorphisms with Ischemic Stroke

To assess the impact of different classes of gamma-glutamyltransferases, we utilized genotyping data on polymorphisms covering not only the *GGT5* and *GGT6* genes but also the *GGT1* and *GGT7* genes, which were previously genotyped in our earlier studies [38,39]. The combined effect of GGT gene family polymorphisms on susceptibility to ischemic stroke was evaluated using diplotype association analysis by comparing the distribution of genotype combinations between case and control groups stratified by sex. Statistical analysis was performed using STATISTICA 13.0. *p*-values were adjusted for multiple testing using the false discovery rate (FDR) method, implemented via the FDR online calculator (https://www.sdmproject.com/utilities/?show=FDR, accessed on 27 October 2025), with adjustments performed separately for men and women.

#### 2.3.4. MB-MDR Analysis of Gene–Gene and Gene–Environment Interactions

The model-based multifactor dimensionality reduction (MB-MDR) approach was used to explore gene–gene and gene–environment interactions associated with ischemic stroke. MB-MDR is an extension of the traditional MDR method [45] and was implemented in the mbmdr package for R, v.2.6 [46]. In contrast to conventional MDR analysis, the MB-MDR approach was developed to detect a set of statistically significant gene–gene (G×G) and gene–environment (G×E) interactions, rather than identifying a single optimal model. The MB-MDR analytical algorithm we used in our previous studies has been described in detail [47,48]. Briefly, each multilocus genotype combination, along with environmental risk factors, was tested for association with IS using logistic regression. Based on these tests, each genotype was classified as high-risk, low-risk, or no evidence of risk. Next, genotypes pooled into high- and low-risk categories were evaluated for association with IS using logistic regression, with significance assessed via Wald statistics (WH, WL). Finally, interaction models were ranked according to empirical *p*-values adjusted for multiple testing using permutations. The MB-MDR analysis was stratified by sex to assess sex-specific effects. Interaction orders 2 through 4, including both G×G (GGT × GGT polymorphisms) and G×E (GGT polymorphisms × regular alcohol use/cigarette smoking) interactions, were evaluated, with age included as a covariate.

#### 2.3.5. Model Stability, Prioritization, and Sensitivity Analysis

**Figure 2:**
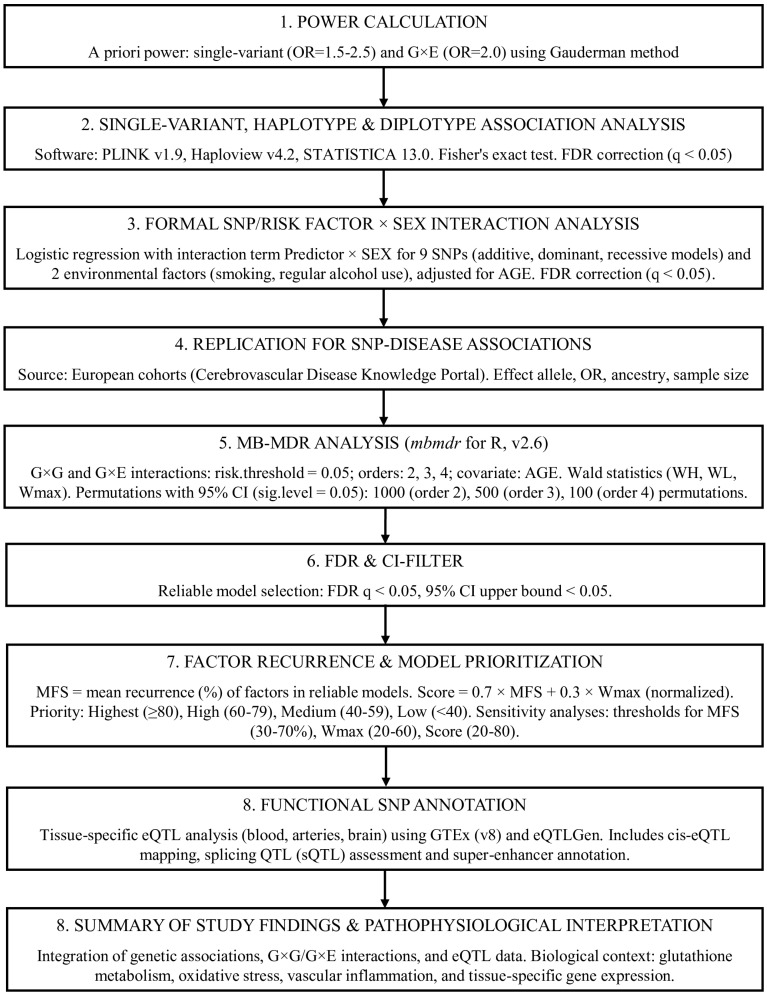
Statistical and bioinformatics analysis pipeline.

Based on our previous experience, MB-MDR tends to identify a relatively large number of statistically significant models, raising concerns about overfitting and unstable cell counts, particularly when some genotype combination cells are rare. To address this issue, we developed an algorithm for selecting and evaluating the most significant models, presented as a pipeline in Figure 2.

The pipeline comprises nine sequential stages: power calculation; single-variant, haplotype and diplotype analyses; formal SNP/factor × sex interaction; replication; MB-MDR analysis; FDR and CI-filtering; factor recurrence and model prioritization; functional SNP annotation; and summary of findings with pathophysiological interpretation. Arrows indicate the flow of analysis. Detailed descriptions of each stage are provided in the Section 2.

First, to enhance robustness against sparse cell classification, we set risk.threshold = 0.05 (default = 0.1), requiring stronger statistical evidence for risk group assignment [49]. For each interaction model, Wald statistics (WH, WL) and the maximum Wald statistic (Wmax = max(WH, WL)) were derived. Second, permutation testing was performed with sig.level = 0.05 to compute 95% confidence intervals (CI) for permutation *p*-values (P_perm_) using the normal approximation [50,51]. To balance computational efficiency with estimation stability, the number of permutations was set to 1000 for second-order interactions, 500 for third-order, and 100 for fourth-order interactions. Models were retained if the upper confidence bound was < 0.05 or P_perm_ = 0 with non-estimable CI. Third, false discovery rate correction was applied separately to each interaction order within each stratum, with q < 0.05 considered statistically significant. Fourth, following MB-MDR analysis with FDR and CI-filtering, we assessed model stability by calculating factor recurrence—the percentage of reliable models (q < 0.05 and CI-filter passed) containing each SNP or environmental factor. For each model, the Mean Factor Stability (MFS) was derived as the average recurrence (%) of its constituent factors. Fifth, to prioritize models, we constructed a weighted composite score combining MFS and normalized Wmax: Score = 0.7 × MFS + 0.3 × Wmax_norm, where MFS is the Mean Factor Stability (in percent, 0–100), and Wmax_norm is the normalized Wald statistic calculated as: Wmax_norm = 100 × (Wmax − min_Wmax)/(max_Wmax − min_Wmax), resulting in a 0–100 scale. Based on the distribution of scores across reliable models, we classified models as Highest (Score ≥ 80), High (60–79), Medium (40–59), or Low (<40) priority. Only Highest and High priority models were presented in the main text. Sixth, robustness was confirmed through sensitivity analyses applying increasingly stringent thresholds for MFS (30–70%), Wmax (20–60), and composite score (20–80). Models were considered stable if they remained significant after threshold application.

#### 2.3.6. Functional SNP Annotation

To evaluate the potential functional consequences of the identified variants, we used the Ensembl genome browser (http://www.ensembl.org/index.html, accessed on 19 October 2025) to classify variants by their genomic location and predicted protein impact. Pathogenicity predictions for missense variants were obtained from Ensembl’s variant effect predictor. Expression quantitative trait locus (eQTL) and splicing QTL (sQTL) data were retrieved from the Genotype-Tissue Expression (GTEx) project (v8) and the eQTLGen consortium. Associations between the analyzed polymorphisms and gene expression levels were examined in whole blood, arterial tissues (aorta, tibial artery, and coronary artery), and brain regions (cortex, cerebellum, and hypothalamus). Significance thresholds were set at *p* < 0.05 after multiple testing correction where applicable.

## 3. Results

### 3.1. Association Analysis Between GGT5 and GGT6 Gene Polymorphisms and Ischemic Stroke

The genotype frequencies of the *GGT5* and *GGT6* gene polymorphisms conformed to Hardy–Weinberg equilibrium in the cohort (*p* > 0.05). Table 1 shows association analysis of *GGT5* and *GGT6* gene polymorphisms with the risk of ischemic stroke in the entire and sex-stratified groups. Polymorphisms rs8140505 and rs2267073 of the *GGT5* gene were associated with increased and decreased risk of ischemic stroke, respectively. These associations were observed in both the entire group and the female subgroup. In addition, the SNP rs2275984 of *GGT5* was also associated with IS risk, but only among females. No significant association was observed between the other examined *GGT6* polymorphisms and ischemic stroke in either the overall cohort or the sex-stratified subgroups.

To formally evaluate whether the observed sex differences were statistically significant, we performed logistic regression with an interaction term (Predictor × SEX) for all SNPs (additive, dominant, and recessive models) and two environmental factors (cigarette smoking and regular alcohol use) while adjusting for age. After FDR correction for 29 tests, only cigarette smoking showed a significant interaction with sex (FDR = 3.38 × 10^−9^, OR = 7.08), while no SNP or regular alcohol use × sex interaction reached significance (all q > 0.05).

### 3.2. Replication of Associations in Independent European Cohorts

Summary statistics obtained from the Cerebrovascular Disease Knowledge Portal (https://cd.hugeamp.org, accessed on 4 October 2025) were used to validate the associations between polymorphisms in the *GGT5* and *GGT6* genes and ischemic stroke in independent cohorts of European ancestry (Supplementary Table S2). Notably, most analyses available in the portal are reported for the combined sample without sex stratification; therefore, replication could only be assessed in a sex-independent manner. In contrast to our findings, the GGT5 variant rs8140505 was associated with an increased risk of large artery atherosclerosis and with small artery occlusion in males. The *GGT5* variant rs2267073 demonstrated a protective effect against large artery atherosclerosis and small artery occlusion in a sex-independent manner in two European cohorts, respectively. Consistent with our findings, none of the examined *GGT6* polymorphisms showed an association with ischemic stroke in the European cohorts analyzed. In contrast to our results, which indicated a positive association between rs2275984 and increased ischemic stroke risk, studies in two European populations reported an association between this variant and decreased disease risk. Thus, the associations of rs8140505 and rs2267073 with increased and decreased stroke risk, respectively, can be considered broadly replicated with respect to the direction of effect for these variants, although the question of their sex-specificity cannot be addressed using the available summary data.

### 3.3. Associations of GGT5 and GGT6 Haplotypes with the Risk of Ischemic Stroke

Table 2 presents the results of the association analysis between haplotypes of the *GGT5* and *GGT6* genes and the risk of ischemic stroke. Five and three common haplotypes, each with a frequency of 5% or higher, have been identified in the *GGT5* and *GGT6* genes, respectively. The analysis of the overall cohort did not reveal a statistically significant correlation between the *GGT5* and *GGT6* haplotypes and susceptibility to ischemic stroke (P_perm_ > 0.05). However, a sex-stratified analysis showed that a common haplotype of *GGT5*—rs8140505A-rs2275984T-rs2267073T—was protective against the risk of ischemic stroke in females (P_perm_ = 0.02). SNP rs2267073 was in negative linkage disequilibrium with both rs8140505 (D = −0.094, D′ = 0.810, *p* < 0.0001) and rs2275984 (D = −0.089, D′ = 0.702, *p* < 0.0001) polymorphisms of the *GGT5* gene. Polymorphisms rs2100986 and rs11657054 of the *GGT6* gene were in positive linkage disequilibrium with each other (D = 0.108, D′ = 0.853, *p* < 0.0001).

Notably, these environmental risk factors are also associated with elevated blood gamma-glutamyl transferase levels and exhibit a dose-dependent and synergistic effect on GGT levels [52,53]. In this context, it was important to investigate whether smoking and regular alcohol use could serve as risk factors that modify the relationship between the analyzed polymorphisms of the *GGT5* and *GGT6* genes and the risk of developing ischemic stroke. To address this issue, we utilized data from a patient survey on lifestyle risk factors, as described earlier [38,39]. To preserve statistical power in evaluating gene–environment interactions, analyses were conducted on the entire cohort, with adjustments for patient sex and age. Table 3 provides a comprehensive summary of the combined effects of smoking, regular alcohol use, and the *GGT5* and *GGT6* polymorphisms on susceptibility to ischemic stroke.

It was found that the rs2267073 polymorphism of the *GGT5* gene exerts a protective effect against the risk of ischemic stroke exclusively in non-smoking individuals, regardless of their sex or age (OR = 0.57 95%CI 0.39–0.85, P_perm_ = 0.005, recessive model). Intricate and compelling patterns emerged from the analysis of the interactions between regular alcohol use and the *GGT5* and *GGT6* polymorphisms in influencing the IS risk. In particular, the protective effect of the rs2267073 polymorphism in the *GGT5* gene against ischemic stroke was also observed in individuals who do not use alcohol regularly (OR = 0.60, 95% CI: 0.39–0.93, P_perm_ = 0.02). Conversely, in those who consume excessive amounts of alcohol, this variant was associated with an increased risk of disease (OR = 8.6, 95% CI: 1.09–67.69, P_perm_ = 0.037). Therefore, this variant has bidirectional effects on the risk of developing ischemic stroke, depending on the presence of individual risk factors such as regular alcohol use and smoking. Paradoxically, the SNP rs2275984 of *GGT5* was associated with the risk of ischemic stroke in individuals who did not drink alcohol regularly, whereas no significant association between this variant and disease risk was observed in those who use alcohol regularly. Furthermore, the polymorphism rs8140505 of *GGT5* conferred protection against IS risk in individuals who abuse alcohol; however, no effect of this SNP on disease risk was observed in those who do not drink alcohol. We also found that rs11657054 of the *GGT6* gene was associated with an increased risk of developing ischemic stroke in individuals who regular alcohol use (OR = 3.79, 95% CI: 1.18–12.14, P_perm_ = 0.02). In the absence of this risk factor, the effect of this variant was neutral with respect to disease development.

### 3.4. Diplotype Analysis of GGT Gene Family Variants

Given the importance of GGT as a risk factor for cerebrovascular disease, our study focused on whether polymorphisms in various genes within the γ-glutamyl transferase family exert a combined effect on ischemic stroke risk. To analyze the role of GGT gene-by-gene interactions in ischemic stroke, we also utilized genotyping data for *GGT1* and *GGT7* gene polymorphisms obtained from our previously published studies [38,39]. To achieve this objective, we conducted a pairwise association analysis examining combinations of *GGT1*, *GGT5*, *GGT6*, and *GGT7* genotypes (diplotypes) in relation to the risk of ischemic stroke. This analysis was performed on the entire cohort as well as on subgroups stratified by sex. Diplotypes of GGT genes that showed associations with ischemic stroke risk, defined by a *p*-value below 0.05, are presented in Supplementary Table S3. A total of 17 and 35 diplotypes were associated with ischemic stroke susceptibility in males and females, respectively. Table 4 summarizes GGT diplotypes that are significantly associated with the risk of ischemic stroke after adjustment for multiple testing using the false discovery rate. Among all groups, 27 GGT diplotypes have shown significant associations with the risk of ischemic stroke. Seven diplotypes were found to be associated with IS susceptibility in females (FDR < 0.05). As shown in Table 4, polymorphisms in the *GGT5* (rs8140505, rs2267073, rs2275984) and *GGT1* (rs5760489, rs5751909, rs4820599) genes, as well as rs11546155 in the *GGT7* gene, exerted the strongest combined effects on the risk of ischemic stroke in females. No statistically significant associations (FDR > 0.05) between GGT diplotypes and ischemic stroke risk were observed in males.

### 3.5. Gene–Gene and Gene–Environment Interactions

Ischemic stroke is a multifactorial polygenic disorder shaped by complex gene–gene and gene–environment interactions. To address the methodological challenges of detecting such high-order interactions, we employed Model-Based Multifactor Dimensionality Reduction with a refined analytical pipeline that incorporated stability assessment and model prioritization, enabling systematic evaluation of sex-specific interaction patterns.

After applying MB-MDR with a stricter risk threshold (0.05) and permutation testing with confidence intervals, followed by FDR correction (q < 0.05), 216 reliable models (models that passed both FDR and CI-filter) were retained in women and 59 in men (Supplementary Table S4).

Factor recurrence analysis revealed striking sex-specific patterns. In women, cigarette smoking was the dominant factor, appearing in 75.9% of all reliable models. In men, the dominant factors were rs5751909 of *GGT1* (81.4%) and regular alcohol use (79.7%), while cigarette smoking appeared in only 25.4% of models (Supplementary Table S4). To prioritize models, we calculated Mean Factor Stability (MFS) as the average recurrence of constituent factors for each model, normalized Wmax to a 0–100 scale, and constructed a weighted composite score: Score = 0.7 × MFS + 0.3 × Wmax_norm. The weights were chosen empirically based on the distribution of MFS and normalized Wmax across reliable models. MFS exhibited lower variability and more consistent biological interpretation across strata, while Wmax varied considerably. Therefore, MFS was assigned a higher weight (0.7) to prioritize factor recurrence breadth without excessively penalizing models with moderate signal strength but high stability. Based on the distribution of scores, models were classified as Highest (Score ≥ 80), High (60–79), Medium (40–59), or Low (<40) priority. Top prioritized MB-MDR models in women and men are shown in Table 5. Among 216 reliable models in women, 12 models achieved High priority (scores 60.1–68.1), including 9 s-order interactions between cigarette smoking and individual *GGT1*, *GGT5*, *GGT6*, and *GGT7* variants, and 3 third-order cigarette smoking × *GGT5/GGT1* variant interactions. Among 59 reliable models in men, 8 models achieved High or Highest priority. The Highest priority model (Score = 86.4) was the second-order interaction regular alcohol use × SNP rs5751909 of the *GGT1* gene. Seven additional models achieved High priority (scores 60.6–73.5), involving second-, third-, and fourth-order interactions centered on rs5751909 of *GGT1* and regular alcohol use (Table 5). Notably, women showed predominantly second-order interactions with cigarette smoking, whereas men exhibited a broader interaction landscape spanning orders 2 through 4, with GGT1 variants serving as a central hub across all prioritized male models.

To assess the robustness of the prioritized models, we performed sensitivity analyses by applying progressively stringent thresholds for the composite Score. When the threshold was increased from 0 to 60, the number of reliable models in women decreased from 23 to 9, while all 9 High priority models remained stable. At Score ≥ 70, no women models retained significance. In men, the number of reliable models decreased from 59 to 8 at Score ≥ 60, with all 8 prioritized models (1 Highest and 7 High) remaining stable. At Score ≥ 70, two models remained (the Highest and one High priority model), while at Score ≥ 80 only the Highest priority model remained significant. Thus, sensitivity analysis confirmed the stability of all 20 models included in Table 5 at the composite Score threshold of 60, which corresponds to the selection criteria for High priority models. The Highest priority model in men demonstrated the greatest robustness, remaining significant even at the most stringent threshold. These results indicate that the prioritized models are not statistical artifacts and possess sufficient stability for further interpretation.

### 3.6. Functional Effects of SNPs

Understanding the pathophysiological mechanisms through which gene polymorphisms contribute to disease development is impossible without evaluating the functional effects of genetic variants at the level of gene products. According to data available on the Ensembl website (http://www.ensembl.org/index.html, accessed on 19 October 2025), polymorphisms rs8140505 and rs2267073 are intronic variants of the *GGT5* gene, whereas SNP rs2275984 is a missense variant (K330R) of *GGT5* with neutral effects at the protein level, as assessed by various pathogenicity prediction tools. The GTEx project and the eQTLGen consortium databases were utilized to evaluate whether the investigated polymorphisms affect the expression levels of specific genes (Supplementary Table S5). The rs2275984C allele is significantly associated with reduced expression levels of the *GGT5* gene in whole blood (*p* = 3.3 × 10^−7^), the aorta (*p* = 0.001), and various brain regions (*p* < 0.01). The rs8140505G and rs2267073T alleles were not found to be associated with *GGT5* expression levels in either whole blood or arterial tissue. However, the rs2267073T allele correlated with decreased expression of *GGT5* across multiple brain regions (*p* < 0.01). Notably, data from the GTEx portal indicate that the rs8140505 variant acts as a splicing quantitative trait locus (sQTL) associated with a significant reduction in splicing events of the *GGT5* and *POM121L9P* genes in fibroblast cells (*p* = 2.4 × 10^−16^). This finding suggests that the variant may disrupt an enhancer element that facilitates a specific splice junction, thereby reducing the proportion of transcripts that include this junction.

As shown in Supplementary Table S5, the ischemic stroke-associated allele rs8140505G is strongly associated (*p* < 1.0 × 10^−15^) with increased expression of multiple genes, including *GSTT1*, *SUSD2*, and *DDT*, as well as decreased expression of *UPB1* in whole blood. In addition, this allele was found to be associated with decreased expression of the *POM121L9P* gene in the tibial artery (*p* < 0.0001). In peripheral blood, the presence of the rs2275984-C allele was significantly associated with reduced expression levels of *GGT5*, *UPB1*, *GSTT1*, *GGT1*, *DDT*, *SUSD2*, and *SPECC1L* (*p* < 0.00001). Additionally, this allele showed a strong positive correlation with increased expression of *ADORA2A* in blood (*p* = 5.2 × 10^−12^) as well as with increased expression of *ADORA2A-AS1* in the tibial artery (*p* = 0.0001). Decreased levels of *POM121L9P* in both arterial and brain tissues were found to be associated with carriage of the rs2275984C and rs2267073T alleles (*p* < 0.0001). Allele rs2267073T is also strongly correlated with increased expression of *UPB1* in blood (*p* = 7.6 × 10^−234^) and increased expression of *ADORA2A-AS1* in the tibial artery (*p* = 0.000005). This allele also exhibited a strong negative correlation with the expression levels of *GSTT1*, *ADORA2A*, *SUSD2*, and *DDT* in the blood (*p* = 1.0 × 10^−7^), as well as with *POM121L9P* in arteries and the brain (*p* < 0.00001). In the coronary artery, the rs2267073T allele is also associated with decreased levels of *GGTLC4P* (*p* = 0.00003).

No significant eQTLs were detected in these tissues for the rs11657054 and rs2100986 polymorphisms of the *GGT6* gene (Supplementary Table S5). Both variant alleles, rs11657054-G and rs2100986-C, were found to be significantly associated with reduced expression of *MYBBP1A* in whole blood, while simultaneously exhibiting increased expression of this gene in multiple arterial tissues and the hypothalamus (*p* < 0.0001). These alleles were also associated with decreased expression of *CXCL16* in the blood (*p* < 0.00001). In whole blood, the allele rs2100986-C showed an association with increased levels of *ALOX15* and decreased levels of *PLD2* (*p* < 0.00001). This allele was also associated with decreased levels of *SMTNL2* mRNA in the tibial artery (*p* = 0.0001).

## 4. Discussion

Population-based studies have shown that elevated gamma-glutamyl transferase levels, independent of alcohol intake, are positively associated with a range of cardiometabolic and lifestyle factors, including advanced age, male sex, higher BMI, smoking, sedentary lifestyle, hypertension, tachycardia, hyperglycemia, hypertriglyceridemia, elevated LDL cholesterol, and decreased HDL cholesterol [12]. Even modestly elevated plasma GGT levels are associated with increased morbidity, mortality, and poorer prognosis of cardiovascular and other chronic diseases [54]. Mendelian randomization studies have suggested that elevated blood GGT, as well as increased levels of alanine aminotransferase (ALT) and aspartate aminotransferase (AST), are associated with a higher incidence of stroke and may be implicated as causal factors in the disease’s pathogenesis [23,24,25]. While gamma-glutamyltransferase is a known risk factor for cerebrovascular disease, polymorphisms in its genes have yet to be systematically studied in ischemic stroke pathogenesis. This pilot study examined the contribution of *GGT5* and *GGT6* gene polymorphisms to ischemic stroke susceptibility, along with a comprehensive assessment of polymorphisms across the full GGT gene family.

This study provides the first evidence of an association between *GGT5* gene polymorphisms and ischemic stroke risk, with marked sexual dimorphism that may be driven by sex-specific environmental modulators such as smoking and regular alcohol consumption. In females, the rs8140505A-rs2275984T-rs2267073T haplotype of *GGT5* was linked to decreased ischemic stroke susceptibility. The replication analysis confirms that rs8140505 and rs2267073 of *GGT5* are consistently associated with ischemic stroke in independent European cohorts, supporting their role as genuine susceptibility loci, although the direction of association for rs2275984 differs between our study (risk increasing in women) and external cohorts (protective), and sex-specific effects could not be evaluated due to the lack of stratified summary statistics. Discrepancies may arise from methodological heterogeneity across studies, unmeasured environmental exposures (smoking and alcohol) that modify genetic effects in a sex-specific manner, genetic ancestry differences, and statistical power limitations. These findings underscore the critical need for sex-stratified analyses in genetic association studies to fully characterize sex-specific genetic architecture.

Our study identified novel gene–environment interactions, revealing that cigarette smoking in women and regular alcohol use in men differentially modulate the effects of GGT family variants on ischemic stroke risk. In particular, the *GGT5* rs2267073 variant was associated with decreased IS risk exclusively in non-smokers and non-drinkers, independent of gender and age. In contrast, among regular alcohol users, this same polymorphism was associated with increased risk of ischemic stroke. Although *GGT6* gene polymorphisms did not show statistically significant associations with ischemic stroke in overall and gender-stratified analyses, the SNP rs11657054 was positively associated with an increased risk of ischemic stroke in individuals who consume alcohol regularly. Unexpectedly, rs8140505 exerted a protective effect against disease susceptibility in regular alcohol drinkers, while rs2275984 was associated with increased risk in non-drinkers. These findings point to a potential modulatory role of alcohol consumption patterns in GGT–stroke associations, warranting confirmation in larger studies with more rigorous alcohol intake assessment.

Diplotype analysis revealed that polymorphisms across GGT genes jointly influence ischemic stroke risk, particularly in females, where seven diplotypes remained significant after multiple testing correction. Risk-associated combinations included *GGT5* genotypes (rs8140505-G/G and rs2275984-T/C) together with *GGT1* variants (rs5760489, rs5751909, and rs4820599) previously linked to stroke in our earlier study [39]. Thus, in females, the risk effect of the heterozygous rs2275984 (T/C) genotype of the *GGT5* gene was more clearly manifested in combination with GGT1 genotypes. Protective diplotypes against ischemic stroke included combinations of *GGT5* rs2267073-T/T with *GGT7* rs11546155-G/G and *GGT5* rs2275984-C/C with *GGT1* rs5751909-G/A—both previously associated with decreased disease risk in our earlier studies [38,39].

To further explore the complexity of genetic interactions, we employed MB-MDR analysis, which revealed intricate G×G and G×E interactions within the GGT gene family influencing ischemic stroke risk. In women, significant interactions were observed between smoking and variants in *GGT1* (rs5751909, rs4820599, rs5760489) and *GGT5* (rs8140505, rs2267073). In men, consistent with earlier findings, *GGT5* variants showed no synergistic effects; instead, the strongest interactions involved regular alcohol use, smoking, and polymorphisms in *GGT1* (rs5751909, rs4820599, rs5760489), *GGT6* (rs2100986, rs11657054), and *GGT7* (rs11546155). Gene–gene interaction analyses—both diplotype-based and MB-MDR—revealed synergistic effects among GGT gene polymorphisms in influencing ischemic stroke risk, supporting a polygenic model of susceptibility and a potential etiological role for GGT isoenzymes. Inclusion of cigarette smoking and regular alcohol use in multilevel MB-MDR models further highlighted the multifactorial nature of the disease. The strongest associations involved *GGT5* and *GGT1* variants, consistent with their physical proximity in the 22q11.23 region. This genomic localization reflect linkage disequilibrium and shared regulatory elements—such as enhancers modulating redox-related gene expression—that could underlie the observed genotype–phenotype relationships.

Functional annotation of the identified variants provides potential mechanistic insights. GGT5 emerges as a compelling candidate for genetic studies of cerebral atherosclerosis, given its elevated expression in arterial tissues, as documented by the GTEx Portal (Supplementary Table S5). Notably, the opposite risk alleles of *GGT5* variants rs2275984 (risk-increasing) and rs2267073 (protective)—which are in negative linkage disequilibrium—are associated with opposing effects on the expression of multiple genes, according to eQTLGen consortium data. Although rs2275984 is a missense variant (Lys→Arg), the C allele—carried by the risk genotype rs2275984T/C—was significantly associated with reduced *GGT5* expression in whole blood, aorta, and multiple brain regions (Supplementary Table S5). This finding may be explained by the fact that the rs2275984 variant is in linkage disequilibrium with a gene variant that affects *GGT5* expression. Although SNP rs2267073 does not correlate with *GGT5* gene expression in the blood or arteries, it is nonetheless associated with gene expression levels in various regions of the brain.

The identification of numerous cis-eQTLs for *GGT5* polymorphisms—associated with expression of blood-based genes including *UPB1*, *GSTT1*, *SUSD2*, *DDT*, *POM121L9P*, *ADORA2A*, *ADORA2A-AS1*, and *GGTLC4P*—suggests that these variants reside within regulatory elements (enhancers or silencers) in the 22q11.23 genomic region. According to the SuperEnhancer database, SNP rs2267073 of *GGT5* is part of a super-enhancer (ID SE_02_201900971) approximately 20 kb in length, located in this region. Notably, a super-enhancer in aortic smooth muscle cells documented in the database may influence atherosclerosis by modulating these interactions. Importantly, the genes whose expression correlates with *GGT5* variants are functionally linked to cellular redox homeostasis, further supporting their relevance in vascular pathology. Importantly, the genes whose expression correlates with polymorphisms of the *GGT5* gene are functionally linked to cellular redox homeostasis, further supporting their pathogenetic relevance in vascular pathology through oxidative stress. This is exemplified by the functional interrelationship among *GGT5*, *GGT1*, and *GSTT1*—all located within the same genomic cluster and centrally involved in glutathione metabolism, a key antioxidant system for combating oxidative stress. Coordinated regulation of these genes by shared transcription factors or chromatin states may enable synchronized activation of cellular antioxidant responses. GSTT1 conjugates glutathione to electrophiles [55], while GGT1 and GGT5 regulate redox homeostasis by supplying cysteine for glutathione synthesis [56]. Thus, reduced GGT5 expression in arterial tissue may impair the hydrolysis of extracellular glutathione, thereby limiting the supply of cysteine for de novo glutathione synthesis in vascular cells [12,13]. This would compromise the antioxidant capacity of the arterial wall, particularly under conditions of increased oxidative stress, such as smoking or alcohol consumption.

The biological roles of genes co-expressed with *GGT5* variants represent a compelling area of interest. *UPB1*, strongly associated with *GGT5* risk variants, encodes β-ureidopropionase, which catalyzes the final step of pyrimidine degradation to produce β-alanine [57]. β-alanine is a precursor of carnosine, a dipeptide antioxidant that scavenges reactive oxygen species, chelates metals, and buffers pH—thereby contributing to glutathione preservation and cellular protection against oxidative stress [58,59]. Reduced UPB1 expression, correlated with the risk alleles of rs8140505 and rs2275984, may impair carnosine synthesis, thereby diminishing systemic antioxidant capacity [60]. *ADORA2A* and *ADORA2A-AS1*, whose expression correlates with *GGT5* risk variants, are involved in purinergic signaling. *ADORA2A* encodes the adenosine A2A receptor, which regulates cardiac rhythm, cerebral circulation, neuroinflammation, and sleep [61,62,63]. Its activation exerts anti-inflammatory effects by inhibiting NF-κB, a master regulator of immune responses, cell proliferation, apoptosis, and stress responses to a variety of noxious stimuli, including oxidative stress [64,65,66]. DDT, a hypoxia-regulated enzyme of the MIF family, has been shown to confer cardioprotection against ischemia–reperfusion injury in mice [67,68]; its downregulation, correlated with the risk alleles of *GGT5*, may thus mirror vulnerability to ischemic damage.

For the *GGT6* variant rs11657054, no significant eQTLs were detected for GGT6 itself, indicating that its risk effect in regular alcohol consumers is likely mediated through pleiotropic regulation of distal genes rather than direct cis-regulation. The risk-associated G allele was correlated to reduced *MYBBP1A* expression in blood but increased *MYBBP1A* expression in multiple arterial tissues and the hypothalamus, alongside increased *CXCL16* expression in blood. MYBBP1A is a transcriptional regulator involved in stress responses [69]; its increase in arterial tissues may predispose to maladaptive vascular stress responses, while its decrease in blood may reflect systemic dysregulation. Elevated *CXCL16*, a chemokine implicated in vascular inflammation, atherosclerosis, and plaque instability, provides a direct pro-atherogenic mechanism [70,71]. In regular alcohol consumers, alcohol metabolism generates oxidative stress and depletes glutathione [52], which may potentiate these effects, tipping the balance toward vascular inflammation and increased ischemic stroke risk.

In women, cigarette smoking emerged as the dominant environmental trigger, interacting exclusively with individual GGT variants through second-order interactions. This pattern suggests that in women, the pathogenic mechanism may be primarily driven by the cumulative effect of smoking combined with single GGT polymorphisms, with no evidence of higher-order epistasis. Biologically, this could reflect a pathway whereby smoking-induced oxidative stress—a process in which gamma-glutamyltransferase enzymes play a central role [12]—is amplified by specific GGT variants. In men, by contrast, the *GGT1* variant rs5751909 served as a central genetic hub, interacting with regular alcohol use and other GGT variants across second-, third-, and fourth-order MB-MDR interactions. The presence of higher-order interactions in men raises the possibility that ischemic stroke susceptibility in this sex may arise from more intricate polygenic interplay, where multiple GGT family members act in concert with lifestyle factors. The rs5751909 variant could represent a regulatory node that modulates the activity of other GGT genes through shared transcriptional or post-transcriptional mechanisms. The involvement of alcohol consumption as a key modifier in men aligns with well-established observations that alcohol affects glutathione metabolism and that GGT is a sensitive biomarker of alcohol intake [12,52].

Sexual dimorphism in ischemic stroke susceptibility is well documented [72,73], and genetic associations with complex diseases frequently exhibit sex specificity beyond X-linked effects [74,75]. The pronounced sexual dimorphism in GGT–stroke associations—particularly in gene–environment interactions—was expected, given sex-specific patterns of smoking and alcohol use [76,77] (established stroke risk factors [78,79,80]) alongside well-documented sex differences in serum GGT levels that are linked to hormonal influences, body mass index, and alcohol consumption [81,82,83]. We propose that much of this dimorphism arises because both smoking and alcohol elevate serum GGT levels through hepatotoxic effects, suggesting that environmental exposures modulate genetic risk in a sex-dependent manner. Several mechanisms may explain sex dimorphism in GGT–disease associations. First, sex hormones are known to differentially regulate hepatic GGT expression and glutathione metabolism [84,85]; estrogens exhibit antioxidant properties that may attenuate vascular damage in women [86]. Second, sex differences in alcohol metabolism [77] are relevant, as alcohol potently induces GGT through hepatocyte injury and enzyme induction [87]. Higher alcohol consumption in men may amplify liver damage, thereby diminishing the capacity for glutathione synthesis. Third, central obesity—more common in men—is more strongly associated with elevated GGT and cardiometabolic risk [88,89]. MB-MDR analysis revealed that the most pronounced sex-specific effects involved GGT–smoking and GGT–alcohol interactions. In men, the absence of *GGT5* interactions and stronger *GGT1/GGT6/GGT7* signals with smoking/alcohol suggest that alternative pathways—such as hepatic detoxification and leukotriene-mediated inflammation—may predominate. These findings underscore sex as a critical biological variable in cerebrovascular genetics.

Multiple mechanisms may underlie the involvement of GGT family genes in the pathogenesis of ischemic stroke. γ-Glutamyltransferase primarily functions to initiate the degradation of extracellular glutathione, glutathione conjugates, and other γ-glutamyl compounds by cleaving their γ-glutamyl bonds, thereby facilitating de novo glutathione biosynthesis [90]. Elevated GGT expression enhances glutathione synthesis, conferring increased resistance to oxidative stress [56,91]. Although GGT and LDL co-localize in atherosclerotic plaques—suggesting possible pro-oxidant effects via GGT-mediated iron reduction [92,93,94]—this does not imply causality. GGT recruitment may instead reflect increased glutathione demand in response to oxidative stress. Moreover, most mechanistic data come from in vitro models, limiting insights into whole-organism adaptive responses. Thus, it remains unclear whether GGT actively promotes LDL oxidation or accumulates passively as part of a protective antioxidant response during atherogenesis [95]. It is plausible that GGT1 and GGT5, both possessing established catalytic activity, contribute to disease development at several levels. During the atherothrombotic phase, these enzymes—localized within the vascular wall and atherosclerotic plaques—may promote oxidative modification of low-density lipoproteins through GGT-mediated iron reduction, potentially exacerbating endothelial dysfunction and vascular inflammation. Additionally, GGT1 and GGT5 might modulate inflammatory responses by converting leukotriene C4 to leukotriene D4, key mediators of vascular permeability and vasoconstriction, thereby influencing atherosclerotic plaque stability and susceptibility to rupture. In the context of ischemic brain injury, genetically determined alterations in GGT activity—by regulating glutathione metabolism and cysteine availability for de novo glutathione synthesis—may affect the capacity of neurons and glial cells to counteract oxidative stress induced by hypoxia and reperfusion. Collectively, polymorphisms in the *GGT1*, *GGT5*, *GGT6*, and *GGT7* genes may constitute an integrative mechanism linking disruptions in redox homeostasis, prostaglandin metabolism, and inflammatory responses, potentially contributing to polygenic susceptibility to cerebrovascular pathology at all stages of progression—from early atherosclerotic changes to irreversible brain tissue damage.

The present study has several limitations. The study investigated a limited subset of functionally relevant GGT gene polymorphisms that may contribute to susceptibility to ischemic stroke. Further genetic association studies with comprehensive coverage of independent polymorphisms influencing gene activity and expression are necessary to accurately assess the overall contribution of GGT genes to susceptibility to ischemic stroke. The associations between *GGT5* and *GGT6* gene polymorphisms and the risk of ischemic stroke observed in our study, including their sex-specific effects, should be validated in other ethnic groups with larger sample sizes. Although formal SNP × sex interaction testing confirmed a significant sex difference for smoking, no individual SNP or alcohol × sex interaction reached significance after multiple testing correction. This may be attributed to limited statistical power for detecting interaction effects in single-variant tests, particularly given the relatively modest sample size after stratification and the stringent correction for multiple tests. Importantly, the absence of significant SNP × sex interactions does not invalidate the sex-specific higher-order interactions identified by MB-MDR, as the latter captures complex, multi-locus effects that are not detectable in single-variant interaction tests. Nevertheless, the lack of formal statistical support for SNP × sex interactions in the single-variant framework suggests that the female-specific associations observed for individual GGT variants should be interpreted with caution and require replication in larger, sex-stratified cohorts. We acknowledge that some diplotype combinations included in the analysis had small cell counts, which may lead to unstable odds ratios and inflated effect estimates. Although we applied FDR correction to mitigate the risk of false-positive findings, this does not fully address the instability arising from low-frequency genotype combinations. Therefore, the results of the diplotype analysis should be interpreted with caution. Technical limitations of the mbmdr package version 2.6 precluded direct extraction of cell sizes, though factor recurrence analysis provided a direct, interpretable measure of stability. The missing data for alcohol consumption and the self-reported nature of exposures may introduce measurement error, potentially attenuating true interaction effects. Binary coding of environmental factors limited exposure granularity, and the single-ancestry population (East Slavic) limits generalizability to other populations. A limitation of the study also relates to the methodology used to assess alcohol consumption. We relied solely on drinking frequency without quantifying the amount consumed per occasion. This approach prevented us from calculating total ethanol intake and, importantly, from identifying binge drinking patterns—a well-established risk factor for stroke. Consequently, our analysis likely underestimates the true association between hazardous alcohol use and disease risk. Therefore, the observed effects should be interpreted as relating to drinking frequency rather than alcohol dose. Our findings show that associations between *GGT5/GGT6* polymorphisms and ischemic stroke risk are modulated by environmental factors, particularly regular alcohol use—a known stroke risk factor that elevates blood GGT levels. Further studies should collect detailed data on smoking and alcohol consumption to clarify dose-dependent effects and gene–environment interactions. Ultimately, functional studies examining GGT enzyme expression and activity in cerebral arteries and brain tissue—beyond blood—are needed to elucidate their role in stroke pathogenesis. Finally, the absence of an independent replication cohort represents the most significant constraint; external validation is essential to confirm these hypothesis-generating findings.

## 5. Conclusions

This is the first study to examine *GGT5* and *GGT6* polymorphisms in relation to ischemic stroke risk and to comprehensively assess the contribution of the entire GGT gene family to disease susceptibility. We identified novel associations for *GGT5* variants rs8140505, rs2275984, and rs2267073, which exhibited marked sexual dimorphism. Additionally, the haplotype rs8140505A–rs2275984T–rs2267073T of *GGT5* was linked to a decreased risk of ischemic stroke in females. Notably, the protective effect of the rs2267073 variant against ischemic stroke risk was observed exclusively among non-smokers who did not regularly consume alcohol, whereas this SNP increased disease risk in individuals who regularly consumed alcohol; these associations were independent of sex. MB-MDR analysis revealed sex-specific gene–gene and gene–environment interactions within the GGT gene family—involving smoking and regular alcohol use—that influence susceptibility to ischemic stroke. In women, the interaction landscape was dominated by second-order interactions between cigarette smoking and individual *GGT5* and *GGT1* variants, suggesting a relatively simple pathogenic mechanism. In men, by contrast, *GGT1* variants emerged as a central genetic hub participating in higher-order interactions with alcohol consumption and other GGT family members (predominantly *GGT6*), indicating a more complex polygenic architecture.

The study findings generate a novel hypothesis regarding the combined involvement of GGT gene family polymorphisms and pro-oxidant environmental factors in the polygenic predisposition to ischemic stroke. By shifting the focus from serum γ-glutamyltransferase as a conventional biochemical risk marker to the genetic architecture of the GGT gene family, this work provides a framework for understanding the molecular underpinnings of disease susceptibility. The pronounced sexual dimorphism observed here underscores the necessity of considering sex as a critical biological variable in genetic association studies of cerebrovascular disease. These results suggest that preventive strategies for ischemic stroke may need to be tailored by sex, with smoking cessation potentially being particularly impactful in women carrying *GGT5* risk variants and alcohol moderation being relevant in men with *GGT1* variants.

Future studies should prioritize independent replication in larger, diverse populations. Prospective studies with detailed quantitative assessment of smoking and alcohol consumption—including dose, duration, and patterns of use—are needed to clarify the dose-dependent nature of the observed gene–environment interactions. Functional investigations using in vitro and in vivo models are essential to elucidate the molecular mechanisms linking GGT variants to glutathione metabolism, oxidative stress, and vascular pathology. Tissue-specific expression studies examining GGT enzyme activity in cerebral arteries and brain parenchyma—rather than peripheral blood alone—will be critical for understanding local pathogenic relevance. Ultimately, integrating genetic, environmental, and functional data may enable the development of sex-specific risk prediction models and inform targeted preventive strategies for ischemic stroke.

## Figures and Tables

**Table 1:** Association analysis of *GGT5* and *GGT6* gene polymorphisms with the risk of ischemic stroke in the entire and sex-stratified groups.

SNP ID	Genotype	N (%)		OR (95% CI) ^1^	P_perm_^2^
		Patients with Ischemic Stroke	Healthy Controls		
Entire groups (600 patients with IS and 688 controls)					
*GGT5* (rs8140505)	A/A	310 (51.7)	366 (53.2)	1.57 (1.03–2.40)	**0.04**
	A/G	237 (39.5)	282 (41.0)		
	G/G	53 (8.8)	40 (5.8)		
*GGT5* (rs2275984)	T/T	286 (47.7)	364 (52.9)	1.23 (0.99–1.54)	**0.05**
	T/C	258 (43.0)	256 (37.2)		
	C/C	56 (9.3)	68 (9.9)		
*GGT5* (rs2267073)	C/C	174 (29.0)	203 (29.5)	0.73 (0.55–0.98)	**0.04**
	C/T	335 (55.8)	350 (50.9)		
	T/T	91 (15.2)	135 (19.6)		
*GGT6* (rs11657054)	A/A	379 (63.2)	463 (67.3)	1.20 (0.95–1.51)	0.09
	A/G	200 (33.3)	204 (29.7)		
	G/G	21 (3.5)	21 (3.1)		
*GGT6* (rs2100986)	T/T	417 (69.5)	502 (73.0)	1.18 (0.93–1.51)	0.15
	T/C	167 (27.8)	173 (25.1)		
	C/C	16 (2.7)	13 (1.9)		
Men (330 patients with IS and 366 controls)					
*GGT5* (rs8140505)	A/A	174 (52.7)	199 (54.4)	1.16 (0.68–1.98)	0.42
	A/G	126 (38.2)	138 (37.7)		
	G/G	30 (9.1)	29 (7.9)		
*GGT5* (rs2275984)	T/T	165 (50.0)	189 (51.6)	0.86 (0.52–1.43)	0.86
	T/C	135 (40.9)	139 (38.0)		
	C/C	30 (9.1)	38 (10.4)		
*GGT5* (rs2267073)	C/C	87 (26.4)	112 (30.6)	1.23 (0.88–1.71)	0.15
	C/T	180 (54.5)	181 (49.5)		
	T/T	63 (19.1)	73 (19.9)		
*GGT6* (rs11657054)	A/A	205 (62.1)	242 (66.1)	1.19 (0.87–1.62)	0.30
	A/G	114 (34.5)	114 (31.1)		
	G/G	11 (3.3)	10 (2.7)		
*GGT6* (rs2100986)	T/T	231 (70.0)	268 (73.2)	1.17 (0.84–1.63)	0.32
	T/C	90 (27.3)	90 (24.6)		
	C/C	9 (2.7)	8 (2.2)		
Women (270 patients with IS and 322 controls)					
*GGT5* (rs8140505)	A/A	136 (50.4)	167 (51.9)	2.63 (1.26–5.51)	**0.02**
	A/G	111 (41.1)	144 (44.7)		
	G/G	23 (8.5)	11 (3.4)		
*GGT5* (rs2275984)	T/T	121 (44.8)	175 (54.3)	1.47 (1.06–2.03)	**0.03**
	T/C	123 (45.6)	117 (36.3)		
	C/C	26 (9.6)	30 (9.3)		
*GGT5* (rs2267073)	C/C	87 (32.2)	91 (28.3)	0.49 (0.30–0.78)	**0.002**
	C/T	155 (57.4)	169 (52.5)		
	T/T	28 (10.4)	62 (19.3)		
*GGT6* (rs11657054)	A/A	174 (64.4)	221 (68.6)	1.21 (0.86–1.70)	0.25
	A/G	86 (31.9)	90 (28.0)		
	G/G	10 (3.7)	11 (3.4)		
*GGT6* (rs2100986)	T/T	186 (68.9)	234 (72.7)	1.20 (0.84–1.71)	0.41
	T/C	77 (28.5)	83 (25.8)		
	C/C	7 (2.6)	5 (1.6)		

^1^ Crude odds ratio with 95% confidence intervals for SNP–disease association; ^2^ *p*-values calculated using permutations with the PLINK 1.9 software. The odds ratio with a 95% confidence interval and the permutation *p*-value for SNP–disease association. Significant permutation *p*-values are bolded.

**Table 2:** Haplotypes of the *GGT5* and *GGT6* genes and their associations with IS risk.

Haplotype		Haplotype Frequencies		Chi Square	P_perm_
		IS Patients	Controls		
Entire groups					
*GGT5*	ATT	0.380	0.402	1.25	0.75
	ATC	0.156	0.167	0.61	0.95
	ACC	0.146	0.135	0.69	0.93
	GTC	0.141	0.136	0.14	0.99
	GCC	0.125	0.111	1.32	0.72
	ACT	0.032	0.033	0.03	1.00
	GTT	0.014	0.010	1.05	0.81
*GGT6*	AT	0.781	0.808	2.93	0.09
	GC	0.148	0.132	1.51	0.22
	GT	0.053	0.047	0.50	0.48
	AC	0.017	0.013	0.83	0.36
Men					
*GGT5*	ATT	0.418	0.399	0.52	0.95
	ATC	0.131	0.164	2.96	0.30
	ACC	0.140	0.135	0.08	0.99
	GTC	0.146	0.137	0.23	0.99
	GCC	0.120	0.118	0.01	0.92
	ACT	0.029	0.035	0.32	0.98
	GTT	-	-	-	-
*GGT6*	AT	0.780	0.807	1.56	0.51
	GC	0.149	0.135	0.62	0.79
	GT	0.057	0.048	0.48	0.89
	AC	0.014	0.010	0.49	0.87
Women					
*GGT5*	ATT	0.333	0.402	6.075	**0.02**
	ATC	0.188	0.173	0.427	0.97
	ACC	0.154	0.136	0.813	0.88
	GTC	0.135	0.135	0.001	1.00
	GCC	0.133	0.102	2.682	0.32
	ACT	0.035	0.031	0.105	0.99
	GTT	0.020	0.015	0.432	0.97
*GGT6*	AT	0.782	0.810	1.36	0.56
	GC	0.147	0.128	0.911	0.77
	GT	0.049	0.046	0.069	0.99
	AC	0.021	0.016	0.397	0.91

The estimation of haplotype frequencies and significance levels for haplotype-disease associations was performed by the Haploview software v.4.2. Significant permutation *p*-values are bolded.

**Table 3:** Joint effects of gamma-glutamyl transferase gene polymorphisms and environmental risk factors on susceptibility to ischemic stroke (gene–environment interactions analysis).

Gene	SNP ^1^	Genotype	Presence of Risk Factor			Absence of Risk Factor		
			Patients with Ischemic Stroke, N (%)	Healthy Controls, N (%)	OR (95% CI) P_perm_^2^	Patients with Ischemic Stroke, N (%)	Healthy Controls, N (%)	OR (95% CI) P_perm_^2^
SNP-cigarette smoking interactions								
*GGT5*	rs8140505	A/A	136 (51.3)	125 (56.6)	1.25	174 (51.9)	241 (51.6)	0.98
		A/G	106 (40.0)	81 (36.7)	(0.86–1.82)	131 (39.1)	201 (43.0)	(0.74–1.30)
		G/G	23 (8.7)	15 (6.8)	0.25	30 (9.0)	25 (5.4)	1.00
	rs2275984	T/T	121 (45.7)	117 (52.9)	1.24	165 (49.3)	247 (52.9)	1.14
		T/C	117 (44.2)	77 (34.8)	(0.86–1.80)	141 (42.1)	179 (38.3)	(0.86–1.51)
		C/C	27 (10.2)	27 (12.2)	0.21	29 (8.7)	41 (8.8)	0.42
	rs2267073	C/C	73 (27.5)	74 (33.5)	1.10	101 (30.1)	129 (27.6)	0.57
		C/T	146 (55.1)	108 (48.9)	(0.68–1.80)	189 (56.4)	242 (51.8)	(0.39–0.85)
		T/T	46 (17.4)	39 (17.6)	0.60	45 (13.4)	96 (20.6)	**0.005** ^R^
*GGT6*	rs11657054	A/A	174 (65.7)	153 (69.2)	1.30	205 (61.2)	310 (66.4)	1.26
		A/G	81 (30.6)	61 (27.6)	(0.87–1.94)	119 (35.5)	143 (30.6)	(0.93–1.84)
		G/G	10 (3.8)	7 (3.2)	0.17	11 (3.3)	14 (3.0)	0.12
	rs2100986 ^D^	T/T	195 (73.6)	163 (73.8)	1.09	222 (66.3)	339 (72.6)	1.34
		T/C	60 (22.6)	52 (23.5)	(0.71–1.67)	107 (31.9)	121 (25.9)	(0.99–1.83)
		C/C	10 (3.8)	6 (2.7)	0.86	6 (1.8)	7 (1.5)	0.059
SNP- regular alcohol use interactions								
*GGT5*	rs8140505	A/A	64 (55.2)	7 (28.0)	0.31	246 (50.8)	131 (58.0)	1.35
		A/G	44 (37.9)	15 (60.0)	(0.12–0.81)	193 (39.9)	82 (36.3)	(0.98–1.86)
		G/G	8 (6.9)	3 (12.0)	**0.016** ^D^	45 (9.3)	13 (5.8)	0.08
	rs2275984	T/T	56 (48.3)	9 (36.0)	0.58	230 (47.5)	130 (57.5)	1.52
		T/C	46 (39.7)	14 (56.0)	(0.23–1.43)	212 (43.8)	81 (35.8)	(1.10–2.10)
		C/C	14 (12.1)	2 (8.0)	0.19	42 (8.7)	15 (6.6)	**0.01** ^D^
	rs2267073	C/C	32 (27.6)	10 (40.0)	8.60	142 (29.3)	72 (31.9)	0.60
		C/T	56 (48.3)	14 (56.0)	(1.09–67.69)	279 (57.6)	112 (49.6)	(0.39–0.93)
		T/T	28 (24.1)	1 (4.0)	**0.037** ^R^	63 (13.0)	42 (18.6)	**0.02**
*GGT6*	rs11657054	A/A	70 (60.3)	21 (84.0)	3.79	309 (63.8)	142 (62.8)	0.95
		A/G	42 (36.2)	3 (12.0)	(1.18–12.14)	158 (32.6)	80 (35.4)	(0.68–1.31)
		G/G	4 (3.4)	1 (4.0)	**0.02** ^D^	17 (3.5)	4 (1.8)	1.00
	rs2100986	T/T	81 (69.8)	22 (88.0)	0.61	336 (69.4)	160 (70.8)	1.03
		T/C	34 (29.3)	3 (12.0)	(0.23–1.62)	133 (27.5)	64 (28.3)	(0.73–1.47)
		C/C	1 (0.9)	0 (0.0)	0.059	15 (3.1)	2 (0.9)	1.00

^1^ Superscript denotes the best genetic model for SNP–disease association: ^D^—dominant, ^R^—recessive; ^2^ the odds ratio with 95% confidence intervals, adjusted for sex and age, along with permutation *p*-value. Bold is statistically significant permutation *p*-values.

**Table 4:** GGT diplotypes significantly associated with the risk of ischemic stroke in both the entire group and among females *.

No.	Diplotypes	Patients with IS		Healthy Controls		OR ^3^	CI^−^	CI^+^	*p*	FDR
		n ^1^	% ^2^	n ^1^	% ^2^					
Entire groups										
1	*GGT6* rs11657054-A/A × *GGT5* rs2275984-C/C	176	29.3	250	36.3	0.73	0.58	0.92	0.008	0.04
2	*GGT6* rs11657054-A/G × *GGT5* rs2267073-C/T	123	20.5	101	14.7	1.50	1.12	2.00	0.006	0.04
3	*GGT6* rs11657054-A/G × *GGT1* rs4820599-A/A	141	23.5	119	17.3	1.47	1.12	1.93	0.006	0.04
4	*GGT6* rs11657054-A/A × *GGT1* rs5751909-G/G	1	0.2	14	2.0	0.12	0.02	0.63	0.0018	0.03
5	*GGT6* rs11657054-A/G × *GGT1* rs5751909-A/A	162	27.0	140	20.3	1.45	1.12	1.88	0.0049	0.04
6	*GGT6* rs2100986-T/T × *GGT1* rs5751909-A/G	86	14.3	139	20.2	0.66	0.49	0.89	0.006	0.04
7	*GGT6* rs2100986-T/T × *GGT1* rs5751909-G/G	1	0.2	15	2.2	0.11	0.02	0.58	0.003	0.04
8	*GGT7* rs11546155-G/G × *GGT5* rs2275984-C/C	218	36.3	296	43.0	0.76	0.60	0.95	0.01	0.04
9	*GGT7* rs11546155-G/A × *GGT5* rs2267073-C/T	81	13.5	59	8.6	1.66	1.17	2.37	0.005	0.04
10	*GGT7* rs11546155-G/G × *GGT1* rs5751909-A/G	82	13.7	149	21.7	0.57	0.43	0.77	0.0002	0.007
11	*GGT5* rs8140505-G/G × *GGT5* rs2275984-C/T	27	4.5	14	2.0	2.27	1.18	4.37	0.01	0.04
12	*GGT5* rs8140505-A/G × *GGT5* rs2267073-T/T	1	0.2	11	1.6	0.15	0.03	0.81	0.008	0.04
13	*GGT5* rs8140505-G/G × *GGT5* rs2267073-C/T	15	2.5	3	0.4	5.18	1.62	16.63	0.004	0.04
14	*GGT5* rs8140505-G/G × *GGT1* rs4820599-A/A	41	6.8	26	3.8	1.87	1.13	3.09	0.01	0.04
15	*GGT5* rs8140505-G/G × *GGT1* rs5760489-A/A	39	6.5	21	3.1	2.21	1.28	3.80	0.01	0.04
16	*GGT5* rs8140505-A/A × *GGT1* rs5751909-A/G	61	10.2	105	15.3	0.63	0.45	0.88	0.006	0.04
17	*GGT5* rs8140505-G/G × *GGT1* rs5751909-A/A	43	7.2	23	3.3	2.23	1.33	3.75	0.002	0.03
18	*GGT5* rs2275984-C/T × *GGT1* rs4820599-A/G	93	15.5	72	10.5	1.57	1.13	2.18	0.007	0.04
19	*GGT5* rs2275984-C/T × *GGT1* rs5760489-A/G	113	18.8	86	12.5	1.62	1.20	2.20	0.002	0.03
20	*GGT5* rs2275984-C/C × *GGT1* rs5751909-A/G	53	8.8	105	15.3	0.54	0.38	0.76	0.0005	0.01
21	*GGT5* rs2275984-C/T × *GGT1* rs5751909-A/A	203	33.8	188	27.3	1.36	1.07	1.73	0.01	0.04
22	*GGT5* rs2267073-C/T × *GGT1* rs5751909-G/G	268	44.7	254	36.9	1.38	1.10	1.72	0.005	0.04
23	*GGT5* rs2267073-T/T × *GGT1* rs5751909-G/A	14	2.3	46	6.7	0.33	0.18	0.61	0.0002	0.007
24	*GGT1* rs4820599-A/A × *GGT1* rs5751909-A/G	58	9.7	104	15.1	0.60	0.43	0.85	0.003	0.04
25	*GGT1* rs5760489-A/A × *GGT1* rs5751909-A/G	49	8.2	85	12.4	0.63	0.44	0.91	0.01	0.04
26	*GGT1* rs5760489-A/G × *GGT1* rs5751909-A/A	170	28.3	132	19.2	1.67	1.28	2.16	0.0001	0.007
27	*GGT1* rs5760489-A/G × *GGT1* rs5751909-A/G	52	8.7	92	13.4	0.61	0.43	0.88	0.008	0.04
Women										
1	*GGT7* rs11546155-G/G × *GGT5* rs2267073-T/T	20	7.4	52	16.1	0.42	0.24	0.72	0.001	0.02
2	*GGT5* rs8140505-G/G × *GGT1* rs5760489-A/A	16	5.9	3	0.9	5.92	1.85	18.98	0.001	0.02
3	*GGT5* rs8140505-G/G × *GGT1* rs5751909-G/G	19	7.0	5	1.6	4.48	1.71	11.70	0.002	0.04
4	*GGT5* rs2275984-C/T × *GGT1* rs4820599-A/G	45	16.7	28	8.7	2.10	1.27	3.47	0.003	0.05
5	*GGT5* rs2275984-C/T × *GGT1* rs5760489-A/G	54	20.0	33	10.2	2.19	1.37	3.49	0.001	0.02
6	*GGT5* rs2275984-C/C × *GGT1* rs5751909-G/A	21	7.8	54	16.8	0.42	0.25	0.71	0.001	0.02
7	*GGT1* rs5760489-A/G × *GGT1* rs5751909-G/G	74	27.4	51	15.8	2.01	1.34	3.00	0.0006	0.02

* No statistically significant associations between GGT diplotypes and the risk of ischemic stroke were observed among males. ^1^ Absolute number of individuals with particular diplotype. ^2^ Percentage of individuals with diplotype. ^3^ OR. odds ratio; 95% CI, confidence intervals. *p*-values were adjusted for multiple testing by false discovery rate (FDR) using FDR online calculator.

**Table 5:** Top Prioritized MB-MDR Models in Women and Men.

Model	Order	NH	NL	Beta_H	Beta_L	Wmax	OR_H	OR_L	P_perm_	MFS (%)	Score	Priority
Men												
ALCOHOL × *GGT1* rs5751909	2	1	2	0.147	−0.253	24.98	1.16	0.78	<0.0001	80.5	86.4	Highest
ALCOHOL × SMOKING × *GGT1* rs5751909	3	1	2	0.151	−0.352	34.38	1.16	0.70	<0.0001	62.2	73.5	High
ALCOHOL × *GGT1* rs5751909 × *GGT1* rs5760489	3	1	4	0.154	−0.295	31.86	1.17	0.74	<0.0001	59.9	68.7	High
ALCOHOL × SMOKING × *GGT1* rs5751909 × *GGT1* rs5760489	4	1	4	0.148	−0.408	43.69	1.16	0.66	<0.0001	51.3	65.9	High
ALCOHOL × *GGT7* rs11546155 × *GGT1* rs5751909	3	1	2	0.163	−0.284	28.12	1.18	0.75	<0.0001	61.0	64.6	High
ALCOHOL × *GGT1* rs5751909 × *GGT6* rs11657054	3	1	4	0.202	−0.264	27.01	1.22	0.77	<0.0001	62.2	64.0	High
ALCOHOL × *GGT1* rs5751909 × *GGT1* rs4820599	3	1	3	0.159	−0.324	27.54	1.17	0.72	<0.0001	59.3	62.7	High
ALCOHOL × *GGT1* rs5751909 × *GGT6* rs2100986	3	1	4	0.249	−0.259	25.98	1.28	0.77	<0.0001	59.3	60.6	High
Women												
SMOKING × *GGT5* rs8140505	2	3	2	0.421	−0.389	68.96	1.52	0.68	<0.0001	54.4	68.1	High
SMOKING × *GGT1* rs5751909	2	2	3	0.421	−0.421	67.85	1.52	0.66	<0.0001	54.9	67.8	High
SMOKING × *GGT1* rs5760489	2	2	1	0.427	−0.209	68.69	1.53	0.81	<0.0001	53.0	67.0	High
SMOKING × *GGT5* rs2275984	2	3	1	0.421	−0.196	67.85	1.52	0.82	<0.0001	53.7	67.0	High
SMOKING × *GGT5* rs2267073	2	3	2	0.421	−0.221	67.85	1.52	0.80	<0.0001	52.3	66.1	High
SMOKING × *GGT1* rs4820599	2	2	1	0.427	−0.201	68.69	1.53	0.82	<0.0001	50.7	65.4	High
SMOKING × *GGT7* rs11546155	2	2	1	0.415	−0.256	64.50	1.51	0.77	<0.0001	50.7	63.3	High
SMOKING × *GGT1* rs5760489 × *GGT5* rs8140505	3	6	2	0.429	−0.237	76.68	1.54	0.79	<0.0001	46.3	62.4	High
SMOKING × *GGT6* rs11657054	2	2	1	0.411	−0.206	62.85	1.51	0.81	<0.0001	49.0	61.3	High
SMOKING × *GGT6* rs2100986	2	2	1	0.411	−0.221	62.85	1.51	0.80	<0.0001	48.9	61.2	High
SMOKING × *GGT1* rs5751909 × *GGT5* rs8140505	3	5	2	0.414	−0.230	71.62	1.51	0.79	<0.0001	47.5	61.0	High
SMOKING × *GGT1* rs5751909 × *GGT5* rs2275984	3	5	1	0.430	−0.298	70.41	1.54	0.74	<0.0001	47.1	60.1	High

NH, number of high-risk genotype cells; NL, number of low-risk genotype cells; β_H, regression coefficient for high-risk group; β_L, regression coefficient for low-risk group; Wmax, maximum Wald statistic; OR_H, odds ratio for high-risk group; OR_L, odds ratio for low-risk group; Pperm, permutation *p*-value; MFS (Mean Factor Stability), average recurrence (%) of constituent factors across all reliable models; Score, weighted composite score = 0.7 × MFS + 0.3 × normalized Wmax (range 0–100). Priority classification: Highest (Score ≥ 80), High (60–79), Medium (40–59), Low (<40). Higher scores indicate greater model robustness and biological relevance.

## Data Availability

The data presented in this study are available upon reasonable request from the corresponding author.

## Supplementary Materials

The following supporting information can be downloaded at: https://www.mdpi.com/article/10.3390/life16050721/s1, Table S1: Quality control of genotyping; Table S2: Validation of the associations between *GGT5* and *GGT6* gene polymorphisms and the risk of ischemic stroke in European populations; Table S3: The GGT diplotypes significantly associated with the risk of ischemic stroke; Table S4: All reliable MB-MDR models in women and men; Table S5: eQTL-analysis of the studied polymorphisms.

## Abbreviations

The following abbreviations are used in this manuscript:

SNP	Single nucleotide polymorphism
IS	Ischemic stroke
GGT	Gamma-glutamyltransferase
MB-MDR	Model-based multifactor dimensionality
eQTL	Expression quantitative trait loci
FDR	False discovery rate
LDL	Low density lipoprotein
