# Supplementary material for: From Serum to Genome: γ-Glutamyltransferase Gene Family Variants Shape Ischemic Stroke Risk via Sex-Specific Gene–Environment Interactions
Source: Life (Basel). 2026 Apr 24;16(5):721. doi: 10.3390/life16050721 (PMC13208725; doi:10.3390/life16050721)
Supplement: Supplementary file 1 [file life-16-00721-s001.zip › Supplementary Table S1.pdf]

Supplementary Table S1 Quality control of genotyping

Gene	SNP	Alleles	Genotype call rate	p-value for HWE in controls
GGT6	rs11657054	A>G	100	0.91
GGT6	rs2100986	T>C	100	0.91
GGT7	rs6119534	C>T	100	<0.0001
GGT7	rs11546155	G>A	100	0.11
GGT5	rs8140505	A>G	100	0.31
GGT5	rs2275984	T>C	100	0.09
GGT5	rs2267073	C>T	100	0.67
GGT1	rs4820599	A>G	100	0.18
GGT1	rs5760489	A>G	100	0.91
GGT1	rs5760492	G>A	100	<0.0001
GGT1	rs5751909	G>A	100	0.64
