# Supplementary material for: From Serum to Genome: γ-Glutamyltransferase Gene Family Variants Shape Ischemic Stroke Risk via Sex-Specific Gene–Environment Interactions
Source: Life (Basel). 2026 Apr 24;16(5):721. doi: 10.3390/life16050721 (PMC13208725; doi:10.3390/life16050721)
Supplement: Supplementary file 1 [file life-16-00721-s001.zip › Supplementary Table S2.pdf]

Supplementary Table S2 Validation of the associations between *GGT5* and *GGT6* gene polymorphisms and the risk of ischemic stroke in European populations (Summary statistics data from the Cerebrovascular Disease Knowledge Portal, https://cd.hugeamp.org)

Phenotype of ischemic stroke	Gene, SNP (effect allele)				
	GGT5			*GGT6*	
	rs8140505 (G)	rs2275984 (C)	rs2267073 (T)	rs11657054 (A)	rs2100986 (T)
*TOAST large artery atherosclerosis♀	♂ OR=1.02 (0.009)[1] (n=1,322)	-	OR=0.86 (0.02)[3] (n=3,612)	-	-
*TOAST small artery occlusion	♂ OR=1.02 (0.02)[1] (n=566)	♂ OR=0.99 (0.024)[1] (n=566)	OR=0.93 (0.036)[4] (n= 27,143)	-	-
Lacunar stroke	-	OR=0.94 (0.016)[2] (n=23,549)	-	-	-
All ischemic stroke	-	-	-	-	-
Small vessel stroke	-	-	-	-	-
Large artery stroke	-	-	-	-	-
Early onset ischemic stroke	-	-	-	-	-

[1]-Ischemic stroke 2024 GWAS: European ancestry males; [2]Lacunar stroke 2021 GWAS: European ancestry; [3]ISGC early-onset ischemic stroke 2022 GWAS: European ancestry; [4]SiGN and ISGC 2016 stroke GWAS: European ancestry.

*According to the TOAST classification (PMID: 7678184), large artery atherosclerosis as an ischemic stroke subtype caused by significant stenosis or occlusion of a major cerebral artery or extracranial vessel. Small-artery occlusion (lacunar stroke) is defined by a stroke resulting from a small vessel disease (typically 1.5 cm or less) in the subcortical white matter or brainstem.
