# Supplementary material for: From Serum to Genome: γ-Glutamyltransferase Gene Family Variants Shape Ischemic Stroke Risk via Sex-Specific Gene–Environment Interactions
Source: Life (Basel). 2026 Apr 24;16(5):721. doi: 10.3390/life16050721 (PMC13208725; doi:10.3390/life16050721)
Supplement: Supplementary file 1 [file life-16-00721-s001.zip › Supplementary Table S3.pdf]

Supplementary Table S3 The GGT diplotypes significantly associated (at a P-value less than 0.05) with the risk of ischemic stroke

№	Diplotypes	Patients with IS		Healthy controls		[3]OR	CI-	CI+	P	FDR
		n[1]	%[2]	n[1]	%[2]					
Entire groups										
1	*GGT6* rs11657054-A/A × *GGT7* rs11546155-A/A	3	0.5	14	2.0	0.27	0.08	0.88	0.03	0.09
2	*GGT6* rs11657054-A/G × *GGT7* rs11546155-G/A	51	8.5	38	5.5	1.59	1.03	2.46	0.04	0.10
3	*GGT6* rs11657054-A/A × *GGT5* rs2275984-C/C	176	29.3	250	36.3	0.73	0.58	0.92	0.008	0.04
4	*GGT6* rs11657054-A/A × *GGT5* rs2275984-C/T	171	28.5	163	23.7	1.28	1.00	1.65	0.05	0.10
5	*GGT6* rs11657054-A/G × *GGT5* rs2267073-C/T	123	20.5	101	14.7	1.50	1.12	2.00	0.006	0.04
6	*GGT6* rs11657054-A/A × *GGT1* rs4820599-A/A	245	40.8	323	46.9	0.78	0.63	0.97	0.03	0.09
7	*GGT6* rs11657054-A/G × *GGT1* rs4820599-A/A	141	23.5	119	17.3	1.47	1.12	1.93	0.006	0.04
8	*GGT6* rs11657054-A/A × *GGT1* rs5760489-A/A	222	37.0	293	42.6	0.79	0.63	0.99	0.04	0.10
9	*GGT6* rs11657054-A/A × *GGT1* rs5751909-A/G	81	13.5	122	17.7	0.72	0.53	0.98	0.04	0.10
10	*GGT6* rs11657054-A/A × *GGT1* rs5751909-G/G	1	0.2	14	2.0	0.12	0.02	0.63	0.0018	0.03
11	*GGT6* rs11657054-A/G × *GGT1* rs5751909-A/A	162	27.0	140	20.3	1.45	1.12	1.88	0.0049	0.04
12	*GGT6* rs2100986-T/C × *GGT5* rs8140505-G/G	19	3.2	8	1.2	2.68	1.19	6.05	0.02	0.07
13	*GGT6* rs2100986-T/T × *GGT5* rs2275984-C/C	199	33.2	264	38.4	0.80	0.63	1.00	0.05	0.10
14	*GGT6* rs2100986-T/C × *GGT5* rs2275984-T/T	21	3.5	12	1.7	2.04	1.00	4.19	0.05	0.10
15	*GGT6* rs2100986-T/C × *GGT1* rs4820599-A/A	119	19.8	105	15.3	1.37	1.03	1.83	0.03	0.09
16	*GGT6* rs2100986-T/T × *GGT1* rs5760489-A/A	241	40.2	315	45.8	0.79	0.64	0.99	0.04	0.10
17	*GGT6* rs2100986-T/T × *GGT1* rs5751909-A/G	86	14.3	139	20.2	0.66	0.49	0.89	0.006	0.04
18	*GGT6* rs2100986-T/T × *GGT1* rs5751909-G/G	1	0.2	15	2.2	0.11	0.02	0.58	0.003	0.04
19	*GGT7* rs11546155-G/G × *GGT5* rs2275984-C/C	218	36.3	296	43.0	0.76	0.60	0.95	0.01	0.04
20	*GGT7* rs11546155-A/A × *GGT5* rs2275984-C/C	1	0.2	9	1.3	0.18	0.03	1.00	0.04	0.10
21	*GGT7* rs11546155-G/A × *GGT5* rs2267073-C/T	81	13.5	59	8.6	1.66	1.17	2.37	0.005	0.04
22	*GGT7* rs11546155-G/A × *GGT1* rs5760489-A/G	56	9.3	43	6.3	1.54	1.02	2.33	0.04	0.10
23	*GGT7* rs11546155-G/G × *GGT1* rs5751909-A/A	367	61.2	375	54.5	1.31	1.05	1.64	0.02	0.07

24	*GGT7* rs11546155-G/G × *GGT1* rs5751909-A/G	82	13.7	149	21.7	0.57	0.43	0.77	0.0002	0.007
25	*GGT7* rs11546155-G/G × *GGT1* rs5751909-G/G	5	0.8	19	2.8	0.32	0.12	0.82	0.02	0.07
26	*GGT5* rs8140505-G/G × *GGT5* rs2275984-C/T	27	4.5	14	2.0	2.27	1.18	4.37	0.01	0.04
27	*GGT5* rs8140505-A/A × *GGT5* rs2267073-C/C	42	7.0	69	10.0	0.68	0.45	1.01	0.05	0.10
28	*GGT5* rs8140505-A/G × *GGT5* rs2267073-T/T	1	0.2	11	1.6	0.15	0.03	0.81	0.008	0.04
29	*GGT5* rs8140505-G/G × *GGT5* rs2267073-C/T	15	2.5	3	0.4	5.18	1.62	16.63	0.004	0.04
30	*GGT5* rs8140505-G/G × *GGT1* rs4820599-A/A	41	6.8	26	3.8	1.87	1.13	3.09	0.01	0.04
31	*GGT5* rs8140505-G/G × *GGT1* rs5760489-A/A	39	6.5	21	3.1	2.21	1.28	3.80	0.01	0.04
32	*GGT5* rs8140505-A/A × *GGT1* rs5751909-A/G	61	10.2	105	15.3	0.63	0.45	0.88	0.006	0.04
33	*GGT5* rs8140505-A/G × *GGT1* rs5751909-G/G	1	0.2	10	1.5	0.16	0.03	0.90	0.03	0.09
34	*GGT5* rs8140505-G/G × *GGT1* rs5751909-A/A	43	7.2	23	3.3	2.23	1.33	3.75	0.002	0.03
35	*GGT5* rs2275984-C/T × *GGT5* rs2267073-C/T	157	26.2	147	21.4	1.30	1.01	1.69	0.04	0.10
36	*GGT5* rs2275984-C/T ×*GGT1* rs4820599-A/G	93	15.5	72	10.5	1.57	1.13	2.18	0.007	0.04
37	*GGT5* rs2275984-C/T × *GGT1* rs5760489-A/G	113	18.8	86	12.5	1.62	1.20	2.20	0.002	0.03
38	*GGT5* rs2275984-C/C × *GGT1* rs5751909-A/G	53	8.8	105	15.3	0.54	0.38	0.76	0.0005	0.01
39	*GGT5* rs2275984-C/C × *GGT1* rs5751909-G/G	3	0.5	13	1.9	0.29	0.09	0.95	0.05	0.10
40	*GGT5* rs2275984-C/T × *GGT1* rs5751909-A/A	203	33.8	188	27.3	1.36	1.07	1.73	0.01	0.04
41	*GGT5* rs2267073-C/T × *GGT1* rs5760489-A/G	130	21.7	119	17.3	1.32	1.00	1.74	0.05	0.10
42	*GGT5* rs2267073-C/T × *GGT1* rs5751909-G/G	268	44.7	254	36.9	1.38	1.10	1.72	0.005	0.04
43	*GGT5* rs2267073-T/T × *GGT1* rs5751909-G/A	14	2.3	46	6.7	0.33	0.18	0.61	0.0002	0.007
44	*GGT1* rs4820599-A/A × *GGT1* rs5760489-A/G	58	9.7	46	6.7	1.49	1.00	2.24	0.05	0.10
45	*GGT1* rs4820599-A/A × *GGT1* rs5751909-A/A	343	57.2	348	50.6	1.30	1.05	1.63	0.025	0.08
46	*GGT1* rs4820599-A/A × *GGT1* rs5751909-A/G	58	9.7	104	15.1	0.60	0.43	0.85	0.003	0.04
47	*GGT1* rs4820599-A/A × *GGT1* rs5751909-G/G	0	0.0	8	1.2	0.07	0.00	1.16	0.02	0.07
48	*GGT1* rs4820599-A/G × *GGT1* rs5751909-A/A	129	21.5	116	16.9	1.35	1.02	1.78	0.03	0.09
49	*GGT1* rs4820599-A/G × *GGT1* rs5751909-A/G	43	7.2	73	10.6	0.65	0.44	0.96	0.03	0.09
50	*GGT1* rs5760489-A/A × *GGT1* rs5751909-A/G	49	8.2	85	12.4	0.63	0.44	0.91	0.01	0.04
51	*GGT1* rs5760489-A/G × *GGT1* rs5751909-A/A	170	28.3	132	19.2	1.67	1.28	2.16	0.0001	0.007
52	*GGT1* rs5760489-A/G × *GGT1* rs5751909-A/G	52	8.7	92	13.4	0.61	0.43	0.88	0.008	0.04
53	*GGT1* rs5760489-A/G × *GGT1* rs5751909-G/G	3	0.5	13	1.9	0.26	0.07	0.92	0.05	0.10

Males									
1	*GGT6* rs11657054-A/G × *GGT1* rs4820599-A/A	81	24.5	65	17.8	1.51	1.04	2.17	0.03	0.29
2	*GGT6* rs11657054-A/A × *GGT1* rs5751909-A/A	1	0.3	9	2.5	0.17	0.03	0.96	0.04	0.29
3	*GGT6* rs11657054-A/G × *GGT1* rs5751909-G/G	94	28.5	78	21.3	1.47	1.04	2.08	0.03	0.29
4	*GGT6* rs2100986-T/C × *GGT1* rs4820599-A/A	65	19.7	52	14.2	1.48	0.99	2.21	0.05	0.32
5	*GGT6* rs2100986-T/C × *GGT1* rs5751909-A/A	1	0.3	9	2.5	0.17	0.03	0.96	0.04	0.29
6	*GGT7* rs11546155-G/A × *GGT5* rs2267073-C/T	47	14.2	34	9.3	1.62	1.01	2.59	0.04	0.29
7	*GGT7* rs11546155-G/G × *GGT1* rs5751909-A/A	206	62.4	193	52.7	1.49	1.10	2.02	0.01	0.27
8	*GGT7* rs11546155-G/G × *GGT1* rs5751909-A/G	44	13.3	78	21.3	0.57	0.38	0.85	0.006	0.27
9	*GGT5* rs8140505-A/A × *GGT5* rs2267073-C/C	16	4.8	36	9.8	0.47	0.25	0.86	0.01	0.27
10	*GGT5* rs8140505-A/A × *GGT1* rs5751909-G/A	29	8.8	54	14.8	0.56	0.35	0.90	0.02	0.29
11	*GGT5* rs2275984-C/T × *GGT1* rs5751909-G/G	111	33.6	98	26.8	1.39	1.00	1.92	0.05	0.32
12	*GGT5* rs2267073-C/T × *GGT1* rs5751909-G/G	148	44.8	134	36.6	1.41	1.04	1.91	0.03	0.29
13	*GGT5* rs2267073-T/T × *GGT1* rs5751909-G/A	8	2.4	27	7.4	0.33	0.15	0.71	0.005	0.27
14	*GGT1* rs4820599-A/A × *GGT1* rs5751909-G/G	190	57.6	180	49.2	1.40	1.04	1.89	0.03	0.29
15	*GGT1* rs4820599-A/A × *GGT1* rs5751909-G/A	29	8.8	52	14.2	0.58	0.36	0.94	0.03	0.29
16	*GGT1* rs5760489-A/G × *GGT1* rs5751909-G/G	96	29.1	81	22.1	1.44	1.02	2.03	0.04	0.29
17	*GGT1* rs5760489-A/G × *GGT1* rs5751909-G/A	26	7.9	48	13.1	0.57	0.34	0.94	0.03	0.29
Females									
1	*GGT6* rs11657054-A/G × *GGT7* rs11546155-G/A	25	9.3	16	5.0	1.95	1.02	3.74	0.04	0.14
2	*GGT6* rs11657054-A/G × *GGT5* rs8140505-G/G	12	4.4	4	1.2	3.42	1.15	10.18	0.04	0.14
3	*GGT6* rs11657054-A/A × *GGT5* rs2275984-C/C	71	26.3	116	36.0	0.63	0.44	0.90	0.01	0.07
4	*GGT6* rs11657054-A/G × *GGT5* rs2267073-C/T	54	20.0	44	13.7	1.58	1.02	2.44	0.04	0.14
5	*GGT6* rs11657054-A/G × *GGT5* rs2267073-T/T	8	3.0	23	7.1	0.41	0.19	0.92	0.04	0.14
6	*GGT6* rs2100986-T/C × *GGT7* rs11546155-G/A	19	7.0	11	3.4	2.14	1.00	4.58	0.05	0.15
7	*GGT6* rs2100986-T/C × *GGT5* rs8140505-G/G	10	3.7	2	0.6	5.17	1.29	20.72	0.02	0.12
8	*GGT6* rs2100986-T/T × *GGT5* rs2267073-T/T	19	7.0	39	12.1	0.55	0.31	0.98	0.04	0.14
9	*GGT6* rs2100986-T/T × *GGT1* rs5751909-G/A	41	15.2	71	22.0	0.63	0.41	0.97	0.03	0.14
10	*GGT7* rs11546155-G/G × *GGT5* rs8140505-A/A	14	5.2	5	1.6	3.26	1.21	8.83	0.02	0.12
11	*GGT7* rs11546155-G/A × *GGT5* rs8140505-G/G	33	12.2	23	7.1	1.81	1.03	3.17	0.04	0.14

12	*GGT7* rs11546155-G/G × *GGT5* rs2275984-C/C	93	34.4	145	45.0	0.64	0.46	0.90	0.01	0.07
13	*GGT7* rs11546155-G/A × *GGT5* rs2275984-C/T	28	10.4	19	5.9	1.85	1.01	3.38	0.05	0.15
14	*GGT7* rs11546155-G/G × *GGT5* rs2267073-T/T	20	7.4	52	16.1	0.42	0.24	0.72	0.001	0.02
15	*GGT7* rs11546155-G/G × *GGT1* rs5751909-G/A	38	14.1	71	22.0	0.58	0.38	0.89	0.01	0.07
16	*GGT7* rs11546155-G/A × *GGT1* rs5751909-G/G	47	17.4	33	10.2	1.85	1.14	2.98	0.01	0.07
17	*GGT5* rs8140505-A/G × *GGT5* rs2275984-C/C	37	13.7	70	21.7	0.57	0.37	0.88	0.01	0.07
18	*GGT5* rs8140505-G/G × *GGT5* rs2275984-C/T	13	4.8	2	0.6	6.72	1.73	26.17	0.03	0.14
19	*GGT5* rs8140505-A/A × *GGT5* rs2275984-T/T	28	10.4	54	16.8	0.57	0.35	0.94	0.02	0.12
20	*GGT5* rs8140505-G/G × *GGT5* rs2275984-C/T	8	3.0	0	0.0	20.89	1.20	363.7	0.006	0.07
21	*GGT5* rs8140505-G/G × *GGT1* rs4820599-A/A	18	6.7	6	1.9	3.57	1.44	8.85	0.006	0.07
22	*GGT5* rs8140505-G/G × *GGT1* rs5760489-A/A	16	5.9	3	0.9	5.92	1.85	18.98	0.001	0.02
23	*GGT5* rs8140505-G/G × *GGT1* rs5751909-G/G	19	7.0	5	1.6	4.48	1.71	11.70	0.002	0.04
24	*GGT5* rs2275984-C/C × *GGT5* rs2267073-T/T	24	8.9	48	14.9	0.56	0.33	0.94	0.03	0.14
25	*GGT5* rs2275984-C/T × *GGT5* rs2267073-C/C	49	18.1	35	10.9	1.82	1.14	2.90	0.01	0.07
26	*GGT5* rs2275984-T/T × *GGT5* rs2267073-C/T	9	3.3	2	0.6	4.66	1.15	18.94	0.03	0.14
27	*GGT5* rs2275984-C/T × *GGT1* rs4820599-A/G	45	16.7	28	8.7	2.10	1.27	3.47	0.003	0.05
28	*GGT5* rs2275984-C/T × *GGT1* rs5760489-A/G	54	20.0	33	10.2	2.19	1.37	3.49	0.001	0.02
29	*GGT5* rs2275984-C/C × *GGT1* rs5751909-G/A	21	7.8	54	16.8	0.42	0.25	0.71	0.001	0.02
30	*GGT5* rs2267073-C/T × *GGT1* rs5760489-A/G	59	21.9	48	14.9	1.60	1.05	2.43	0.03	0.14
31	*GGT5* rs2267073-C/C × *GGT1* rs5751909-G/G	70	25.9	62	19.3	1.47	1.00	2.16	0.05	0.15
32	*GGT5* rs2267073-T/T × *GGT1* rs5751909-G/A	6	2.2	19	5.9	0.38	0.16	0.94	0.04	0.14
33	*GGT1* rs4820599-A/A × *GGT1* rs5760489-A/G	28	10.4	19	5.9	1.85	1.01	3.38	0.05	0.15
34	*GGT1* rs4820599-A/G × *GGT1* rs5751909-G/G	56	20.7	45	14.0	1.61	1.05	2.48	0.03	0.14
35	*GGT1* rs5760489-A/G × *GGT1* rs5751909-G/G	74	27.4	51	15.8	2.01	1.34	3.00	0.0006	0.02

[1]Absolute number of individuals with particular diplotype.
[2]Percentage of individuals with diplotype. [3]OR. odds ratio; 95% CI, confidence intervals.
P-values were adjusted for multiple testing by false discovery rate (FDR) using FDR online calculator (https://www.sdmproject.com/utilities/?show=FDR).
