# Supplementary material for: From Serum to Genome: γ-Glutamyltransferase Gene Family Variants Shape Ischemic Stroke Risk via Sex-Specific Gene–Environment Interactions
Source: Life (Basel). 2026 Apr 24;16(5):721. doi: 10.3390/life16050721 (PMC13208725; doi:10.3390/life16050721)
Supplement: Supplementary file 1 [file life-16-00721-s001.zip › Supplementary Table S4.pdf]

Supplementary Table S4 All (n=275) reliable MB-MDR models in women and men

Model	Order	NH	NL	BetaH	BetaL	Wmax	OR_H	OR_L	Pperm	FDR	MFS (%)	Score (%)	Priority
Men													
ALCOHOL x rs5751909	2	1	2	0.147	-0.253	24.98	1.16	0.78	<0.0001	<0.0001	80.5	86.4	Highest
ALCOHOL x SMOKING x rs5751909	3	1	2	0.151	-0.352	34.38	1.16	0.7	<0.0001	<0.0001	62.2	73.5	High
ALCOHOL x rs5751909 x rs5760489	3	1	4	0.154	-0.295	31.86	1.17	0.74	<0.0001	<0.0001	59.9	68.7	High
ALCOHOL x SMOKING x rs5751909 x rs5760489	4	1	4	0.148	-0.408	43.69	1.16	0.66	<0.0001	<0.0001	51.3	65.9	High
ALCOHOL x rs11546155 x rs5751909	3	1	2	0.163	-0.284	28.12	1.18	0.75	<0.0001	<0.0001	61	64.6	High
ALCOHOL x rs5751909 x rs11657054	3	1	4	0.202	-0.264	27.01	1.22	0.77	<0.0001	<0.0001	62.2	64	High
ALCOHOL x rs5751909 x rs4820599	3	1	3	0.159	-0.324	27.54	1.17	0.72	<0.0001	<0.0001	59.3	62.7	High
ALCOHOL x rs5751909 x rs2100986	3	1	4	0.249	-0.259	25.98	1.28	0.77	<0.0001	<0.0001	59.3	60.6	High
ALCOHOL x SMOKING x rs5751909 x rs11657054	4	1	4	0.249	-0.362	36.07	1.28	0.7	<0.0001	<0.0001	53	58.8	Medium
ALCOHOL x rs5751909 x rs2275984	3	2	4	0.16	-0.255	24.34	1.17	0.77	<0.0001	<0.0001	59.3	58.5	Medium
ALCOHOL x SMOKING x rs5751909 x rs2267073	4	1	5	0.235	-0.353	33.65	1.26	0.7	<0.0001	<0.0001	53	56.2	Medium
ALCOHOL x SMOKING x rs11546155 x rs5751909	4	1	2	0.167	-0.369	33.94	1.18	0.69	<0.0001	<0.0001	52.1	55.9	Medium
ALCOHOL x SMOKING x rs5751909 x rs4820599	4	1	4	0.168	-0.366	34.63	1.18	0.69	<0.0001	<0.0001	50.9	55.8	Medium
ALCOHOL x rs5751909 x rs8140505	3	1	3	0.197	-0.294	21.36	1.22	0.75	<0.0001	<0.0001	60.5	55.4	Medium
ALCOHOL x SMOKING x rs5751909 x rs2100986	4	1	3	0.289	-0.392	33.89	1.34	0.68	<0.0001	<0.0001	50.9	54.9	Medium
ALCOHOL x SMOKING x rs5751909 x rs2275984	4	2	4	0.203	-0.353	33.65	1.23	0.7	<0.0001	<0.0001	50.9	54.7	Medium
ALCOHOL x rs11546155 x rs5751909 x rs5760489	4	2	4	0.188	-0.321	33.77	1.21	0.73	<0.0001	<0.0001	50.4	54.5	Medium
ALCOHOL x rs11546155 x rs5751909 x rs4820599	4	1	3	0.162	-0.392	33.89	1.18	0.68	<0.0001	<0.0001	50	54.4	Medium
ALCOHOL x rs5751909 x rs4820599 x rs11657054	4	1	4	0.19	-0.402	32.77	1.21	0.67	<0.0001	<0.0001	50.9	53.7	Medium
ALCOHOL x rs11546155 x rs5751909 x rs11657054	4	1	4	0.205	-0.299	30.56	1.23	0.74	<0.0001	<0.0001	52.1	52.2	Medium
ALCOHOL x rs5751909 x rs5760489 x rs11657054	4	1	4	0.186	-0.386	30.8	1.2	0.68	<0.0001	<0.0001	51.3	51.9	Medium
ALCOHOL x SMOKING x rs5751909 x rs8140505	4	2	4	0.212	-0.347	29.88	1.24	0.71	<0.0001	<0.0001	51.7	51.2	Medium
ALCOHOL x rs5751909 x rs4820599 x rs2275984	4	1	5	0.237	-0.344	31.3	1.27	0.71	<0.0001	<0.0001	48.7	50.6	Medium
ALCOHOL x rs11546155 x rs5751909 x rs2100986	4	1	4	0.288	-0.291	29.32	1.33	0.75	<0.0001	<0.0001	50	49.4	Medium
ALCOHOL x rs5751909 x rs5760489 x rs4820599	4	1	4	0.154	-0.347	29.88	1.17	0.71	<0.0001	<0.0001	49.1	49.4	Medium
ALCOHOL x rs5751909 x rs2267073 x rs11657054	4	1	4	0.228	-0.534	27.28	1.26	0.59	<0.0001	<0.0001	53	49.3	Medium
ALCOHOL x rs5751909 x rs5760489 x rs2275984	4	1	5	0.229	-0.354	27.87	1.26	0.7	<0.0001	<0.0001	49.1	47.2	Medium

rs5751909 x rs2267073	2	1	2	0.085	-0.291	12.99	1.09	0.75	8.0e-03	0.02	53.4	46.5	Medium
ALCOHOL x rs11546155 x rs5751909 x rs2275984	4	1	4	0.232	-0.282	26.49	1.26	0.75	<0.0001	<0.0001	50	46.3	Medium
ALCOHOL x rs5751909 x rs4820599 x rs2100986	4	1	4	0.247	-0.361	26.61	1.28	0.7	<0.0001	<0.0001	48.7	45.6	Medium
ALCOHOL x rs5751909 x rs2267073 x rs2275984	4	2	5	0.165	-0.349	24.35	1.18	0.71	<0.0001	<0.0001	50.9	44.6	Medium
ALCOHOL x rs5751909 x rs2267073 x rs8140505	4	1	5	0.244	-0.394	23.4	1.28	0.67	<0.0001	<0.0001	51.7	44.2	Medium
ALCOHOL x rs11546155 x rs5751909 x rs2267073	4	2	3	0.225	-0.367	22.06	1.25	0.69	<0.0001	<0.0001	52.1	43	Medium
ALCOHOL x rs5751909 x rs2100986 x rs11657054	4	1	2	0.244	-0.585	22.5	1.28	0.56	<0.0001	<0.0001	50.9	42.6	Medium
rs11546155 x rs5751909	2	1	2	0.099	-0.16	11.35	1.1	0.85	6.0e-03	0.02	51.7	42.5	Medium
ALCOHOL x rs5751909 x rs5760489 x rs2100986	4	1	4	0.247	-0.335	22.78	1.28	0.72	<0.0001	<0.0001	49.1	41.7	Medium
ALCOHOL x rs5751909 x rs8140505 x rs11657054	4	1	4	0.286	-0.345	20.96	1.33	0.71	<0.0001	<0.0001	51.7	41.5	Medium
ALCOHOL x rs5751909 x rs2267073 x rs2100986	4	1	3	0.285	-0.636	20.52	1.33	0.53	<0.0001	<0.0001	50.9	40.4	Medium
SMOKING x rs5751909 x rs2267073	3	0	4	NA	-0.323	17.99	NA	0.72	<0.0001	<0.0001	44.1	39.6	Low
ALCOHOL x rs5751909 x rs4820599 x rs8140505	4	1	4	0.192	-0.389	20.6	1.21	0.68	<0.0001	<0.0001	49.6	39.6	Low
ALCOHOL x rs8140505	2	1	1	0.197	-0.109	9.84	1.22	0.9	1.1e-02	0.02	50	38.6	Low
ALCOHOL x rs11657054	2	1	1	0.22	-0.098	8.71	1.25	0.91	1.3e-02	0.02	52.5	38.5	Low
ALCOHOL x rs5751909 x rs5760489 x rs2267073	4	2	3	0.215	-0.623	17.66	1.24	0.54	<0.0001	<0.0001	51.3	37.6	Low
SMOKING x rs5751909	2	1	1	0.087	-0.403	7.75	1.09	0.67	1.9e-02	0.03	53.4	37.4	Low
ALCOHOL x rs5751909 x rs5760489 x rs8140505	4	1	4	0.164	-0.34	17.69	1.18	0.71	<0.0001	<0.0001	50	36.8	Low
ALCOHOL x rs2100986	2	1	0	0.26	NA	9.29	1.3	NA	1.4e-02	0.02	48.3	36.5	Low
ALCOHOL x rs5751909 x rs2275984 x rs8140505	4	1	4	0.22	-0.318	16.8	1.25	0.73	<0.0001	<0.0001	49.6	35.5	Low
ALCOHOL x rs11546155 x rs8140505	3	2	1	0.223	-0.107	14.72	1.25	0.9	<0.0001	<0.0001	40.7	33	Low
SMOKING x rs5751909 x rs5760489 x rs11657054	4	2	4	0.193	-0.32	20.92	1.21	0.73	<0.0001	<0.0001	37.7	31.7	Low
rs11546155 x rs5751909 x rs2267073	3	2	2	0.153	-0.289	11.26	1.17	0.75	1.4e-02	0.02	42.9	30.1	Low
ALCOHOL x rs8140505 x rs11657054	3	2	0	0.268	NA	11.54	1.31	NA	1.4e-02	0.02	41.8	29.6	Low
rs5751909 x rs2275984 x rs11657054	3	2	2	0.141	-0.26	11.59	1.15	0.77	2.2e-02	0.02	41.2	29.3	Low
rs5751909 x rs5760489 x rs2275984	3	2	0	0.188	NA	11.34	1.21	NA	2.6e-02	0.03	39	27.4	Low
ALCOHOL x SMOKING x rs11546155 x rs2100986	4	1	2	0.285	-0.183	16.63	1.33	0.83	<0.0001	<0.0001	36	25.8	Low
ALCOHOL x rs2267073 x rs2275984 x rs2100986	4	0	2	NA	-0.3	16.07	NA	0.74	<0.0001	<0.0001	34.7	24.3	Low
SMOKING x rs2267073 x rs8140505 x rs11657054	4	0	2	NA	-0.454	19.6	NA	0.64	<0.0001	<0.0001	24.1	20.7	Low
rs11546155 x rs4820599 x rs2267073 x rs11657054	4	3	1	0.231	-0.38	17.97	1.26	0.68	<0.0001	<0.0001	22.4	17.8	Low
SMOKING x rs2267073 x rs8140505	3	0	1	NA	-0.321	11.61	NA	0.73	2.2e-02	0.02	23.7	17.1	Low
rs4820599 x rs2267073 x rs11657054	3	1	0	0.208	NA	12.29	1.23	NA	1.8e-02	0.02	22.6	17.1	Low

Women													
SMOKING x rs8140505	2	3	2	0.421	-0.389	68.96	1.52	0.68	<0.0001	<0.0001	54.4	68.1	High
SMOKING x rs5751909	2	2	3	0.421	-0.421	67.85	1.52	0.66	<0.0001	<0.0001	54.9	67.8	High
SMOKING x rs5760489	2	2	1	0.427	-0.209	68.69	1.53	0.81	<0.0001	<0.0001	53	67	High
SMOKING x rs2275984	2	3	1	0.421	-0.196	67.85	1.52	0.82	<0.0001	<0.0001	53.7	67	High
SMOKING x rs2267073	2	3	2	0.421	-0.221	67.85	1.52	0.8	<0.0001	<0.0001	52.3	66.1	High
SMOKING x rs4820599	2	2	1	0.427	-0.201	68.69	1.53	0.82	<0.0001	<0.0001	50.7	65.4	High
SMOKING x rs11546155	2	2	1	0.415	-0.256	64.5	1.51	0.77	<0.0001	<0.0001	50.7	63.3	High
SMOKING x rs5760489 x rs8140505	3	6	2	0.429	-0.237	76.68	1.54	0.79	<0.0001	<0.0001	46.3	62.4	High
SMOKING x rs11657054	2	2	1	0.411	-0.206	62.85	1.51	0.81	<0.0001	<0.0001	49	61.3	High
SMOKING x rs2100986	2	2	1	0.411	-0.221	62.85	1.51	0.8	<0.0001	<0.0001	48.9	61.2	High
SMOKING x rs5751909 x rs8140505	3	5	2	0.414	-0.23	71.62	1.51	0.79	<0.0001	<0.0001	47.5	61	High
SMOKING x rs5751909 x rs2275984	3	5	1	0.43	-0.298	70.41	1.54	0.74	<0.0001	<0.0001	47.1	60.1	High
SMOKING x rs2267073 x rs8140505	3	6	3	0.435	-0.24	71.3	1.54	0.79	<0.0001	<0.0001	45.8	59.6	Medium
SMOKING x rs5751909 x rs4820599 x rs8140505	4	9	4	0.445	-0.224	82.07	1.56	0.8	<0.0001	<0.0001	42	59.4	Medium
SMOKING x rs5751909 x rs5760489	3	4	3	0.427	-0.275	68.69	1.53	0.76	<0.0001	<0.0001	46.6	59	Medium
SMOKING x rs5760489 x rs2275984	3	5	2	0.429	-0.196	67.86	1.54	0.82	<0.0001	<0.0001	45.8	58.1	Medium
SMOKING x rs5751909 x rs4820599	3	4	3	0.427	-0.252	68.69	1.53	0.78	<0.0001	<0.0001	45.1	57.9	Medium
SMOKING x rs5760489 x rs2267073	3	6	3	0.427	-0.231	68.69	1.53	0.79	<0.0001	<0.0001	44.9	57.8	Medium
SMOKING x rs5751909 x rs5760489 x rs8140505	4	8	5	0.439	-0.285	75.68	1.55	0.75	<0.0001	<0.0001	43.2	57.3	Medium
SMOKING x rs5751909 x rs2267073	3	5	2	0.42	-0.273	65.31	1.52	0.76	<0.0001	<0.0001	46.1	57.1	Medium
SMOKING x rs5760489 x rs4820599	3	3	1	0.427	-0.213	68.69	1.53	0.81	<0.0001	<0.0001	43.8	57	Medium
SMOKING x rs4820599 x rs2275984	3	5	2	0.429	-0.193	67.86	1.54	0.82	<0.0001	<0.0001	44.3	57	Medium
SMOKING x rs2275984 x rs8140505	3	6	2	0.426	-0.21	63.71	1.53	0.81	<0.0001	<0.0001	46.8	56.8	Medium
SMOKING x rs5751909 x rs5760489 x rs4820599	4	6	3	0.413	-0.26	76.56	1.51	0.77	<0.0001	<0.0001	41.3	56.4	Medium
SMOKING x rs11546155 x rs5751909	3	4	2	0.415	-0.232	64.5	1.51	0.79	<0.0001	<0.0001	45.1	56	Medium
SMOKING x rs11546155 x rs5760489	3	4	1	0.42	-0.196	65.31	1.52	0.82	<0.0001	<0.0001	43.8	55.5	Medium
SMOKING x rs11546155 x rs4820599	3	4	1	0.42	-0.175	65.31	1.52	0.84	<0.0001	<0.0001	42.3	54.4	Medium
SMOKING x rs4820599 x rs2267073	3	5	4	0.417	-0.218	63.65	1.52	0.8	<0.0001	<0.0001	43.4	54.4	Medium
SMOKING x rs5760489 x rs11657054	3	4	1	0.417	-0.185	63.65	1.52	0.83	<0.0001	<0.0001	42.7	54	Medium
SMOKING x rs5760489 x rs2100986	3	4	1	0.417	-0.191	63.65	1.52	0.83	<0.0001	<0.0001	42.6	53.9	Medium
SMOKING x rs4820599 x rs8140505	3	4	2	0.42	-0.224	60.44	1.52	0.8	<0.0001	<0.0001	44.8	53.9	Medium

SMOKING x rs5751909 x rs2275984 x rs8140505	4	9	3	0.436	-0.241	66.31	1.55	0.79	<0.0001	<0.0001	43.5	53.3	Medium
SMOKING x rs11546155 x rs2275984	3	5	2	0.405	-0.201	59.62	1.5	0.82	<0.0001	<0.0001	44.3	53.2	Medium
SMOKING x rs5760489 x rs2267073 x rs8140505	4	9	4	0.439	-0.265	68.01	1.55	0.77	<0.0001	<0.0001	41.9	53	Medium
SMOKING x rs4820599 x rs11657054	3	4	1	0.417	-0.189	63.65	1.52	0.83	<0.0001	<0.0001	41.2	52.9	Medium
SMOKING x rs2267073 x rs2275984	3	5	4	0.413	-0.245	57.24	1.51	0.78	<0.0001	<0.0001	45.4	52.9	Medium
SMOKING x rs4820599 x rs2100986	3	4	1	0.417	-0.192	63.65	1.52	0.83	<0.0001	<0.0001	41.1	52.8	Medium
SMOKING x rs11546155 x rs2267073	3	5	3	0.405	-0.221	59.62	1.5	0.8	<0.0001	<0.0001	43.4	52.6	Medium
SMOKING x rs5751909 x rs11657054	3	3	3	0.402	-0.206	58.04	1.49	0.81	<0.0001	<0.0001	44	52.3	Medium
SMOKING x rs5751909 x rs2100986	3	3	3	0.402	-0.221	58.04	1.49	0.8	<0.0001	<0.0001	43.8	52.2	Medium
SMOKING x rs2267073 x rs11657054	3	5	3	0.411	-0.229	60.4	1.51	0.8	<0.0001	<0.0001	42.3	52.1	Medium
SMOKING x rs11546155 x rs8140505	3	4	3	0.401	-0.287	55.65	1.49	0.75	<0.0001	<0.0001	44.8	51.7	Medium
SMOKING x rs5751909 x rs2267073 x rs8140505	4	8	2	0.426	-0.242	63.71	1.53	0.79	<0.0001	<0.0001	42.8	51.7	Medium
SMOKING x rs5751909 x rs5760489 x rs2275984	4	8	5	0.42	-0.251	62.84	1.52	0.78	<0.0001	<0.0001	42.8	51.3	Medium
SMOKING x rs5760489 x rs4820599 x rs2275984	4	6	3	0.426	-0.257	66.16	1.53	0.77	<0.0001	<0.0001	40.8	51.3	Medium
SMOKING x rs11546155 x rs11657054	3	4	2	0.405	-0.197	59.62	1.5	0.82	<0.0001	<0.0001	41.2	51	Medium
SMOKING x rs4820599 x rs2275984 x rs8140505	4	9	3	0.448	-0.189	64.29	1.57	0.83	<0.0001	<0.0001	41.5	51	Medium
SMOKING x rs2267073 x rs2100986	3	5	3	0.404	-0.214	57.21	1.5	0.81	<0.0001	<0.0001	42.1	50.6	Medium
SMOKING x rs8140505 x rs2100986	3	4	2	0.403	-0.219	54.87	1.5	0.8	<0.0001	<0.0001	43.5	50.5	Medium
SMOKING x rs2100986 x rs11657054	3	3	1	0.407	-0.197	58.8	1.5	0.82	<0.0001	<0.0001	40	49.8	Medium
SMOKING x rs8140505 x rs11657054	3	4	2	0.394	-0.205	52.59	1.48	0.81	<0.0001	<0.0001	43.7	49.6	Medium
SMOKING x rs11546155 x rs2100986	3	3	2	0.398	-0.198	56.47	1.49	0.82	<0.0001	<0.0001	41.1	49.5	Medium
SMOKING x rs5760489 x rs4820599 x rs8140505	4	5	2	0.408	-0.242	61.23	1.5	0.79	<0.0001	<0.0001	41.1	49.3	Medium
SMOKING x rs2275984 x rs11657054	3	4	2	0.394	-0.205	52.59	1.48	0.81	<0.0001	<0.0001	43.2	49.2	Medium
SMOKING x rs2275984 x rs2100986	3	4	2	0.394	-0.192	52.59	1.48	0.83	<0.0001	<0.0001	43.1	49.1	Medium
SMOKING x rs5760489 x rs4820599 x rs2100986	4	5	1	0.414	-0.203	62.01	1.51	0.82	<0.0001	<0.0001	38.3	47.8	Medium
SMOKING x rs4820599 x rs2267073 x rs8140505	4	7	4	0.424	-0.248	57.4	1.53	0.78	<0.0001	<0.0001	40.8	47.4	Medium
SMOKING x rs5751909 x rs4820599 x rs2267073	4	7	4	0.407	-0.26	56.43	1.5	0.77	<0.0001	<0.0001	41	47.1	Medium
SMOKING x rs11546155 x rs5760489 x rs4820599	4	4	1	0.407	-0.201	58.8	1.5	0.82	<0.0001	<0.0001	39.2	47	Medium
SMOKING x rs2267073 x rs8140505 x rs11657054	4	8	3	0.413	-0.232	57.24	1.51	0.79	<0.0001	<0.0001	39.9	46.7	Medium
SMOKING x rs5760489 x rs8140505 x rs11657054	4	7	3	0.407	-0.224	56.43	1.5	0.8	<0.0001	<0.0001	40.3	46.6	Medium
SMOKING x rs11546155 x rs5760489 x rs8140505	4	7	3	0.413	-0.223	54.94	1.51	0.8	<0.0001	<0.0001	41.1	46.5	Medium
SMOKING x rs5751909 x rs4820599 x rs2275984	4	6	2	0.41	-0.304	53.4	1.51	0.74	<0.0001	<0.0001	41.7	46.2	Medium

SMOKING x rs5760489 x rs2275984 x rs8140505	4	7	2	0.437	-0.174	51.91	1.55	0.84	<0.0001	<0.0001	42.6	46.2	Medium
SMOKING x rs5760489 x rs4820599 x rs2267073	4	5	3	0.401	-0.218	55.65	1.49	0.8	<0.0001	<0.0001	40	46.1	Medium
SMOKING x rs5760489 x rs2100986 x rs11657054	4	6	1	0.413	-0.186	59.6	1.51	0.83	<0.0001	<0.0001	37.5	46.1	Medium
SMOKING x rs5751909 x rs2275984 x rs11657054	4	5	1	0.456	-0.327	54.16	1.58	0.72	<0.0001	<0.0001	40.9	46	Medium
SMOKING x rs2267073 x rs8140505 x rs2100986	4	7	3	0.42	-0.215	55.82	1.52	0.81	<0.0001	<0.0001	39.8	46	Medium
SMOKING x rs11546155 x rs5751909 x rs5760489	4	5	3	0.4	-0.229	53.33	1.49	0.8	<0.0001	<0.0001	41.3	45.9	Medium
SMOKING x rs11546155 x rs5751909 x rs4820599	4	5	3	0.403	-0.208	54.87	1.5	0.81	<0.0001	<0.0001	40.2	45.8	Medium
SMOKING x rs11546155 x rs5760489 x rs2100986	4	6	2	0.404	-0.203	57.21	1.5	0.82	<0.0001	<0.0001	38.3	45.6	Medium
SMOKING x rs11546155 x rs5751909 x rs2275984	4	6	2	0.397	-0.329	51.81	1.49	0.72	<0.0001	<0.0001	41.7	45.5	Medium
ALCOHOL x SMOKING	2	2	1	0.297	-0.25	31	1.35	0.78	<0.0001	<0.0001	49	45.4	Medium
SMOKING x rs5751909 x rs5760489 x rs11657054	4	5	4	0.4	-0.226	53.33	1.49	0.8	<0.0001	<0.0001	40.5	45.4	Medium
SMOKING x rs5760489 x rs2267073 x rs2275984	4	8	1	0.406	-0.247	51.87	1.5	0.78	<0.0001	<0.0001	41.5	45.4	Medium
SMOKING x rs11546155 x rs5751909 x rs11657054	4	6	3	0.395	-0.281	54.92	1.48	0.76	<0.0001	<0.0001	39.4	45.3	Medium
SMOKING x rs11546155 x rs5751909 x rs8140505	4	6	3	0.393	-0.308	50.31	1.48	0.73	<0.0001	<0.0001	42	45.1	Medium
SMOKING x rs5751909 x rs5760489 x rs2267073	4	6	3	0.403	-0.253	50.35	1.5	0.78	<0.0001	<0.0001	42.1	45.1	Medium
SMOKING x rs11546155 x rs8140505 x rs2100986	4	6	4	0.395	-0.254	54.92	1.48	0.78	<0.0001	<0.0001	39	45	Medium
SMOKING x rs5760489 x rs4820599 x rs11657054	4	4	1	0.401	-0.2	55.65	1.49	0.82	<0.0001	<0.0001	38.4	45	Medium
SMOKING x rs11546155 x rs4820599 x rs2100986	4	6	3	0.404	-0.215	57.21	1.5	0.81	<0.0001	<0.0001	37.2	44.8	Medium
SMOKING x rs5751909 x rs2267073 x rs11657054	4	6	4	0.403	-0.289	52.58	1.5	0.75	<0.0001	<0.0001	40.1	44.8	Medium
SMOKING x rs5751909 x rs2275984 x rs2100986	4	6	1	0.397	-0.34	51.81	1.49	0.71	<0.0001	<0.0001	40.8	44.8	Medium
SMOKING x rs5751909 x rs2267073 x rs2275984	4	7	3	0.392	-0.275	48.09	1.48	0.76	<0.0001	<0.0001	42.5	44.4	Medium
SMOKING x rs5751909 x rs2100986 x rs11657054	4	4	3	0.397	-0.197	54.11	1.49	0.82	<0.0001	<0.0001	38.4	44.3	Medium
SMOKING x rs11546155 x rs5751909 x rs2267073	4	6	4	0.387	-0.281	49.59	1.47	0.76	<0.0001	<0.0001	41	44	Medium
SMOKING x rs11546155 x rs5751909 x rs2100986	4	5	3	0.388	-0.299	51.88	1.47	0.74	<0.0001	<0.0001	39.2	43.8	Medium
SMOKING x rs5751909 x rs4820599 x rs2100986	4	4	2	0.397	-0.189	51.81	1.49	0.83	<0.0001	<0.0001	39.2	43.8	Medium
SMOKING x rs5760489 x rs8140505 x rs2100986	4	6	2	0.403	-0.207	50.35	1.5	0.81	<0.0001	<0.0001	40.2	43.8	Medium
SMOKING x rs2267073 x rs2100986 x rs11657054	4	6	4	0.403	-0.219	54.87	1.5	0.8	<0.0001	<0.0001	37.1	43.7	Medium
SMOKING x rs11546155 x rs4820599 x rs8140505	4	6	3	0.403	-0.208	50.35	1.5	0.81	<0.0001	<0.0001	40	43.6	Medium
SMOKING x rs2275984 x rs8140505 x rs11657054	4	6	2	0.455	-0.19	49.12	1.58	0.83	<0.0001	<0.0001	40.6	43.5	Medium
SMOKING x rs4820599 x rs2267073 x rs2275984	4	7	2	0.411	-0.244	49.03	1.51	0.78	<0.0001	<0.0001	40.4	43.3	Medium
SMOKING x rs5751909 x rs5760489 x rs2100986	4	4	4	0.389	-0.242	48.83	1.48	0.79	<0.0001	<0.0001	40.4	43.2	Medium
SMOKING x rs5751909 x rs4820599 x rs11657054	4	4	2	0.393	-0.186	50.31	1.48	0.83	<0.0001	<0.0001	39.4	43.2	Medium

SMOKING x rs11546155 x rs8140505 x rs11657054	4	6	4	0.393	-0.248	50.31	1.48	0.78	<0.0001	<0.0001	39.1	43	Medium
SMOKING x rs11546155 x rs4820599 x rs2267073	4	7	4	0.393	-0.221	50.31	1.48	0.8	<0.0001	<0.0001	38.9	42.9	Medium
SMOKING x rs11546155 x rs2267073 x rs8140505	4	6	4	0.389	-0.238	46.63	1.48	0.79	<0.0001	<0.0001	40.8	42.5	Medium
SMOKING x rs2267073 x rs2275984 x rs8140505	4	6	2	0.419	-0.224	44.38	1.52	0.8	<0.0001	<0.0001	42.2	42.5	Medium
SMOKING x rs4820599 x rs2100986 x rs11657054	4	4	1	0.397	-0.19	51.81	1.49	0.83	<0.0001	<0.0001	36.4	41.8	Medium
SMOKING x rs2275984 x rs8140505 x rs2100986	4	6	2	0.408	-0.177	45.46	1.5	0.84	<0.0001	<0.0001	40.5	41.8	Medium
SMOKING x rs8140505 x rs2100986 x rs11657054	4	5	2	0.389	-0.2	48.83	1.48	0.82	<0.0001	<0.0001	38.2	41.7	Medium
SMOKING x rs5751909 x rs2267073 x rs2100986	4	5	4	0.396	-0.279	45.25	1.49	0.76	<0.0001	<0.0001	40	41.4	Medium
SMOKING x rs5760489 x rs2275984 x rs11657054	4	5	3	0.408	-0.215	45.46	1.5	0.81	<0.0001	<0.0001	39.9	41.4	Medium
SMOKING x rs4820599 x rs2275984 x rs11657054	4	5	4	0.412	-0.234	46.94	1.51	0.79	<0.0001	<0.0001	38.8	41.3	Medium
SMOKING x rs11546155 x rs4820599 x rs2275984	4	5	3	0.408	-0.203	45.46	1.5	0.82	<0.0001	<0.0001	39.6	41.2	Medium
SMOKING x rs2267073 x rs2275984 x rs2100986	4	6	5	0.376	-0.252	45.24	1.46	0.78	<0.0001	<0.0001	39.5	41	Medium
SMOKING x rs11546155 x rs5760489 x rs11657054	4	5	2	0.38	-0.202	46.67	1.46	0.82	<0.0001	<0.0001	38.4	40.9	Medium
SMOKING x rs11546155 x rs5760489 x rs2267073	4	6	4	0.381	-0.247	43.76	1.46	0.78	<0.0001	<0.0001	40	40.7	Medium
SMOKING x rs11546155 x rs4820599 x rs11657054	4	5	2	0.383	-0.193	48.12	1.47	0.82	<0.0001	<0.0001	37.3	40.7	Medium
SMOKING x rs4820599 x rs8140505 x rs2100986	4	5	2	0.396	-0.205	45.25	1.49	0.81	<0.0001	<0.0001	39	40.7	Medium
SMOKING x rs11546155 x rs2267073 x rs2275984	4	6	2	0.385	-0.31	43.06	1.47	0.73	<0.0001	<0.0001	40.4	40.6	Medium
SMOKING x rs5760489 x rs2275984 x rs2100986	4	5	2	0.372	-0.182	43.83	1.45	0.83	<0.0001	<0.0001	39.8	40.6	Medium
SMOKING x rs11546155 x rs2267073 x rs2100986	4	6	4	0.382	-0.231	45.92	1.47	0.79	<0.0001	<0.0001	38	40.2	Medium
SMOKING x rs5751909 x rs8140505 x rs2100986	4	4	2	0.374	-0.209	40.96	1.45	0.81	<0.0001	<0.0001	41.1	40.2	Medium
SMOKING x rs11546155 x rs2275984 x rs2100986	4	5	2	0.37	-0.191	44.6	1.45	0.83	<0.0001	<0.0001	38.7	40.1	Medium
SMOKING x rs11546155 x rs2100986 x rs11657054	4	4	2	0.383	-0.187	48.12	1.47	0.83	<0.0001	<0.0001	36.4	40.1	Medium
SMOKING x rs2275984 x rs2100986 x rs11657054	4	4	3	0.396	-0.226	45.25	1.49	0.8	<0.0001	<0.0001	37.9	39.8	Low
SMOKING x rs5751909 x rs8140505 x rs11657054	4	4	2	0.363	-0.192	38.96	1.44	0.83	<0.0001	<0.0001	41.2	39.3	Low
SMOKING x rs5760489 x rs2267073 x rs11657054	4	5	3	0.381	-0.266	41.66	1.46	0.77	<0.0001	<0.0001	39.2	39.2	Low
SMOKING x rs4820599 x rs8140505 x rs11657054	4	5	1	0.381	-0.215	41.66	1.46	0.81	<0.0001	<0.0001	39.1	39.1	Low
SMOKING x rs11546155 x rs5760489 x rs2275984	4	4	4	0.389	-0.246	38.37	1.48	0.78	<0.0001	<0.0001	40.8	38.8	Low
SMOKING x rs4820599 x rs2275984 x rs2100986	4	3	5	0.396	-0.256	41.02	1.49	0.77	<0.0001	<0.0001	38.7	38.5	Low
ALCOHOL x SMOKING x rs5760489	3	3	1	0.294	-0.122	28.27	1.34	0.89	<0.0001	<0.0001	42.7	37.8	Low
SMOKING x rs11546155 x rs2267073 x rs11657054	4	5	4	0.367	-0.243	40.31	1.44	0.78	<0.0001	<0.0001	38.1	37.8	Low
SMOKING x rs11546155 x rs2275984 x rs8140505	4	4	2	0.377	-0.191	34.31	1.46	0.83	<0.0001	<0.0001	41.5	37.4	Low
SMOKING x rs2267073 x rs2275984 x rs11657054	4	4	4	0.436	-0.259	37.02	1.55	0.77	<0.0001	<0.0001	39.6	37.3	Low

ALCOHOL x SMOKING x rs4820599	3	3	1	0.296	-0.166	28.99	1.34	0.85	<0.0001	<0.0001	41.2	37.1	Low
SMOKING x rs4820599 x rs2267073 x rs2100986	4	4	3	0.38	-0.2	37.6	1.46	0.82	<0.0001	<0.0001	38	36.5	Low
SMOKING x rs4820599 x rs2267073 x rs11657054	4	4	4	0.369	-0.247	37.55	1.45	0.78	<0.0001	<0.0001	38.1	36.5	Low
ALCOHOL x SMOKING x rs5751909	3	2	2	0.281	-0.24	22.38	1.32	0.79	<0.0001	<0.0001	44	36	Low
SMOKING x rs11546155 x rs2275984 x rs11657054	4	3	2	0.443	-0.2	35.35	1.56	0.82	<0.0001	<0.0001	38.8	36	Low
SMOKING x rs5760489 x rs2267073 x rs2100986	4	3	3	0.363	-0.234	32.3	1.44	0.79	<0.0001	<0.0001	39.1	34.9	Low
ALCOHOL x SMOKING x rs8140505	3	2	2	0.266	-0.193	18.61	1.3	0.82	<0.0001	<0.0001	43.7	34	Low
ALCOHOL x SMOKING x rs2275984	3	2	1	0.275	-0.2	18.91	1.32	0.82	<0.0001	<0.0001	43.2	33.8	Low
ALCOHOL x SMOKING x rs2267073	3	2	1	0.278	-0.334	19.52	1.32	0.72	<0.0001	<0.0001	42.3	33.5	Low
ALCOHOL x SMOKING x rs5751909 x rs2275984	4	2	3	0.261	-0.384	26.65	1.3	0.68	<0.0001	<0.0001	40.9	33.5	Low
ALCOHOL x SMOKING x rs11546155	3	2	1	0.276	-0.211	21.1	1.32	0.81	<0.0001	<0.0001	41.2	33.4	Low
ALCOHOL x SMOKING x rs11657054	3	2	1	0.274	-0.136	20.46	1.32	0.87	<0.0001	<0.0001	40.1	32.4	Low
ALCOHOL x SMOKING x rs2100986	3	2	1	0.274	-0.158	20.46	1.32	0.85	<0.0001	<0.0001	40	32.3	Low
ALCOHOL x SMOKING x rs2267073 x rs8140505	4	2	3	0.28	-0.362	25.16	1.32	0.7	<0.0001	<0.0001	39.9	32.2	Low
ALCOHOL x SMOKING x rs5751909 x rs8140505	4	2	4	0.249	-0.215	21.14	1.28	0.81	<0.0001	<0.0001	41.2	31.3	Low
ALCOHOL x SMOKING x rs5760489 x rs4820599	4	3	1	0.285	-0.137	25.46	1.33	0.87	<0.0001	<0.0001	38.4	31.3	Low
ALCOHOL x SMOKING x rs11546155 x rs5751909	4	2	3	0.26	-0.298	21.39	1.3	0.74	<0.0001	<0.0001	39.4	30.1	Low
ALCOHOL x SMOKING x rs2275984 x rs8140505	4	1	2	0.275	-0.211	19.31	1.32	0.81	<0.0001	<0.0001	40.6	30.1	Low
ALCOHOL x SMOKING x rs5751909 x rs2267073	4	2	3	0.271	-0.334	18.92	1.31	0.72	<0.0001	<0.0001	40.1	29.6	Low
ALCOHOL x SMOKING x rs5760489 x rs2275984	4	3	2	0.295	-0.199	17.17	1.34	0.82	<0.0001	<0.0001	39.9	28.6	Low
ALCOHOL x SMOKING x rs11546155 x rs8140505	4	3	2	0.251	-0.21	18.24	1.29	0.81	<0.0001	<0.0001	39.1	28.5	Low
ALCOHOL x SMOKING x rs11546155 x rs11657054	4	3	2	0.256	-0.214	21.88	1.29	0.81	<0.0001	<0.0001	36.5	28.3	Low
rs5751909 x rs2267073 x rs2275984 x rs8140505	4	4	0	0.529	NA	29.3	1.7	NA	<0.0001	<0.0001	31.7	28.3	Low
ALCOHOL x SMOKING x rs11546155 x rs5760489	4	2	2	0.233	-0.197	18.33	1.26	0.82	<0.0001	<0.0001	38.4	28.1	Low
ALCOHOL x SMOKING x rs11546155 x rs2267073	4	1	2	0.216	-0.334	18.92	1.24	0.72	<0.0001	<0.0001	38.1	28.1	Low
ALCOHOL x SMOKING x rs4820599 x rs2267073	4	3	3	0.263	-0.334	18.92	1.3	0.72	<0.0001	<0.0001	38.1	28.1	Low
ALCOHOL x SMOKING x rs2267073 x rs2275984	4	2	2	0.307	-0.332	16.41	1.36	0.72	<0.0001	<0.0001	39.6	28	Low
ALCOHOL x SMOKING x rs5751909 x rs4820599	4	2	3	0.265	-0.299	16.51	1.3	0.74	<0.0001	<0.0001	39.4	27.9	Low
ALCOHOL x SMOKING x rs5760489 x rs2100986	4	3	2	0.266	-0.14	18.61	1.3	0.87	<0.0001	<0.0001	37.5	27.6	Low
ALCOHOL x SMOKING x rs2267073 x rs11657054	4	3	3	0.265	-0.334	18.92	1.3	0.72	<0.0001	<0.0001	37.2	27.5	Low
rs2267073 x rs2275984 x rs8140505	3	3	1	0.484	-0.123	23.47	1.62	0.88	<0.0001	<0.0001	31	27.4	Low
ALCOHOL x SMOKING x rs5751909 x rs2100986	4	2	2	0.257	-0.268	16.35	1.29	0.76	<0.0001	<0.0001	38.4	27.2	Low

ALCOHOL x SMOKING x rs4820599 x rs2275984	4	2	2	0.272	-0.192	15.88	1.31	0.83	<0.0001	<0.0001	38.8	27.2	Low
rs5751909 x rs5760489 x rs2267073 x rs8140505	4	5	1	0.334	-0.309	27.21	1.4	0.73	<0.0001	<0.0001	31.4	27.2	Low
ALCOHOL x SMOKING x rs11546155 x rs2100986	4	3	2	0.266	-0.166	18.61	1.3	0.85	<0.0001	<0.0001	36.4	26.8	Low
ALCOHOL x SMOKING x rs5760489 x rs11657054	4	3	0	0.259	NA	16.81	1.3	NA	<0.0001	<0.0001	37.6	26.8	Low
ALCOHOL x SMOKING x rs4820599 x rs2100986	4	3	2	0.266	-0.169	18.61	1.3	0.84	<0.0001	<0.0001	36.4	26.8	Low
ALCOHOL x SMOKING x rs2275984 x rs2100986	4	2	2	0.24	-0.194	16.44	1.27	0.82	<0.0001	<0.0001	37.9	26.8	Low
ALCOHOL x SMOKING x rs11546155 x rs4820599	4	3	1	0.259	-0.152	16.81	1.3	0.86	<0.0001	<0.0001	37.3	26.6	Low
ALCOHOL x SMOKING x rs4820599 x rs11657054	4	3	1	0.264	-0.139	18	1.3	0.87	<0.0001	<0.0001	36.5	26.5	Low
ALCOHOL x rs5751909 x rs2267073 x rs8140505	4	0	5	NA	-0.707	28.78	NA	0.49	<0.0001	<0.0001	29.4	26.5	Low
rs5751909 x rs5760489 x rs2275984	3	1	3	0.221	-0.323	19.88	1.25	0.72	<0.0001	<0.0001	31.8	26.3	Low
rs5751909 x rs5760489 x rs8140505	3	2	0	0.257	NA	18.58	1.29	NA	<0.0001	<0.0001	32.3	26	Low
ALCOHOL x rs5751909 x rs8140505	3	1	2	0.275	-0.699	18.53	1.32	0.5	<0.0001	<0.0001	29.6	24.2	Low
rs5751909 x rs5760489	2	1	1	0.172	-0.295	12	1.19	0.74	8.0e-03	0.01	31.9	24	Low
rs5751909 x rs2275984	2	0	1	NA	-0.202	10.89	NA	0.82	1.2e-02	0.02	32.6	23.9	Low
rs5751909 x rs5760489 x rs4820599 x rs8140505	4	2	0	0.41	NA	21.16	1.51	NA	<0.0001	<0.0001	30.6	23.9	Low
rs5760489 x rs8140505	2	1	0	0.399	NA	11.99	1.49	NA	7.0e-03	0.01	31.5	23.7	Low
rs11546155 x rs5751909 x rs5760489 x rs2275984	4	2	4	0.209	-0.349	21.11	1.23	0.71	<0.0001	<0.0001	30.2	23.6	Low
rs2275984 x rs8140505	2	1	1	0.421	-0.135	10.61	1.52	0.87	6.0e-03	0.01	32.2	23.5	Low
rs5751909 x rs5760489 x rs4820599 x rs2275984	4	2	3	0.221	-0.359	20.44	1.25	0.7	<0.0001	<0.0001	30.2	23.3	Low
rs11546155 x rs5751909 x rs5760489 x rs8140505	4	3	1	0.47	-0.19	19.46	1.6	0.83	<0.0001	<0.0001	30.6	23.1	Low
rs11546155 x rs5751909 x rs2275984	3	0	2	NA	-0.245	14.69	NA	0.78	<0.0001	<0.0001	30.3	22.9	Low
rs5760489 x rs2275984	2	1	0	0.193	NA	11.32	1.21	NA	6.0e-03	0.01	30.8	22.8	Low
rs5760489 x rs2267073 x rs2275984 x rs8140505	4	3	0	0.342	NA	18.07	1.41	NA	<0.0001	<0.0001	30.8	22.6	Low
rs5751909 x rs2267073 x rs8140505	3	2	0	0.35	NA	11.54	1.42	NA	3.2e-02	0.03	31.8	22.5	Low
rs5751909 x rs2267073 x rs2275984	3	2	1	0.214	-0.166	12.03	1.24	0.85	2.2e-02	0.02	31.3	22.4	Low
rs2267073 x rs2275984	2	2	1	0.184	-0.14	11.05	1.2	0.87	1.7e-02	0.02	30.1	22.2	Low
rs2267073 x rs8140505	2	1	2	0.551	-0.171	9.8	1.73	0.84	1.4e-02	0.02	30.8	22.1	Low
rs5751909 x rs5760489 x rs2267073	3	2	1	0.218	-0.321	12.17	1.24	0.73	1.8e-02	0.02	30.9	22.1	Low
ALCOHOL x rs5751909 x rs5760489 x rs2275984	4	1	3	0.15	-0.437	19.19	1.16	0.65	<0.0001	<0.0001	29.4	22.1	Low
ALCOHOL x rs5751909 x rs5760489 x rs8140505	4	1	3	0.263	-0.699	18.53	1.3	0.5	<0.0001	<0.0001	29.8	22.1	Low
rs5760489 x rs2267073 x rs2275984 x rs2100986	4	4	0	0.319	NA	21.13	1.38	NA	<0.0001	<0.0001	28	22.1	Low
ALCOHOL x rs5751909 x rs4820599 x rs8140505	4	1	5	0.263	-0.357	20.2	1.3	0.7	<0.0001	<0.0001	28.6	22	Low

rs5760489 x rs4820599 x rs2275984	3	3	0	0.214	NA	14.55	1.24	NA	1.0e-02	0.01	29	21.9	Low
rs4820599 x rs2267073 x rs2275984 x rs8140505	4	3	0	0.465	NA	18.15	1.59	NA	<0.0001	<0.0001	29.6	21.9	Low
rs5760489 x rs2267073 x rs8140505	3	2	2	0.399	-0.133	11.99	1.49	0.88	1.4e-02	0.02	30.6	21.8	Low
rs5751909 x rs4820599 x rs2275984	3	1	1	0.184	-0.329	11.82	1.2	0.72	1.6e-02	0.02	30.3	21.6	Low
rs2267073 x rs8140505 x rs11657054	3	3	0	0.555	NA	14.93	1.74	NA	1.2e-02	0.01	27.9	21.3	Low
ALCOHOL x rs11546155 x rs5751909 x rs8140505	4	0	2	NA	-0.699	18.53	NA	0.5	<0.0001	<0.0001	28.6	21.3	Low
rs11546155 x rs8140505	2	2	0	0.195	NA	10.21	1.22	NA	1.2e-02	0.02	29.2	21.2	Low
ALCOHOL x rs5751909 x rs2275984 x rs8140505	4	0	4	NA	-0.338	16.1	NA	0.71	<0.0001	<0.0001	30.1	21.2	Low
rs5760489 x rs4820599 x rs8140505	3	1	0	0.399	NA	11.99	1.49	NA	1.0e-02	0.01	29.5	21.1	Low
rs11546155 x rs5751909 x rs5760489	3	2	1	0.169	-0.169	11.31	1.18	0.84	2.2e-02	0.02	29.8	21	Low
rs5751909 x rs5760489 x rs8140505 x rs11657054	4	2	0	0.495	NA	15.72	1.64	NA	<0.0001	<0.0001	29.8	20.8	Low
ALCOHOL x rs5751909 x rs8140505 x rs11657054	4	1	3	0.323	-0.699	18.53	1.38	0.5	<0.0001	<0.0001	27.8	20.7	Low
rs5751909 x rs2267073 x rs2275984 x rs2100986	4	2	3	0.423	-0.328	16.6	1.53	0.72	<0.0001	<0.0001	28.9	20.7	Low
ALCOHOL x rs5751909 x rs8140505 x rs2100986	4	0	3	NA	-0.699	18.53	NA	0.5	<0.0001	<0.0001	27.7	20.6	Low
rs4820599 x rs8140505	2	1	0	0.306	NA	8.81	1.36	NA	2.8e-02	0.03	29.2	20.5	Low
ALCOHOL x rs11546155 x rs5751909 x rs2275984	4	0	3	NA	-0.322	17.09	NA	0.72	<0.0001	<0.0001	28.2	20.4	Low
rs5760489 x rs4820599 x rs2275984 x rs11657054	4	4	0	0.283	NA	18.45	1.33	NA	<0.0001	<0.0001	27.3	20.4	Low
rs11546155 x rs2275984	2	1	2	0.152	-0.124	9.05	1.16	0.88	1.7e-02	0.02	28.5	20.1	Low
rs11546155 x rs2267073	2	0	1	NA	-0.203	10.66	NA	0.82	1.6e-02	0.02	27.1	19.9	Low
rs4820599 x rs2275984	2	1	0	0.183	NA	8.73	1.2	NA	3.2e-02	0.04	28.5	19.9	Low
rs5760489 x rs8140505 x rs11657054	3	1	0	0.552	NA	11.07	1.74	NA	2.6e-02	0.03	28.4	19.9	Low
rs11546155 x rs8140505 x rs2100986	3	2	0	0.408	NA	13.19	1.5	NA	1.2e-02	0.01	26.7	19.7	Low
ALCOHOL x rs2267073 x rs8140505	3	0	2	NA	-0.235	11.01	NA	0.79	1.4e-02	0.02	27.9	19.6	Low
ALCOHOL x rs5751909 x rs2275984 x rs11657054	4	0	4	NA	-0.332	16.41	NA	0.72	<0.0001	<0.0001	27.4	19.5	Low
ALCOHOL x rs2267073	2	0	1	NA	-0.235	11.01	NA	0.79	5.0e-03	0.01	25.4	19	Low

NH, number of high-risk genotype cells; NL, number of low-risk genotype cells; β_H, regression coefficient for high-risk group; β_L, regression coefficient for low-risk group; Wmax, maximum Wald statistic; OR_H, odds ratio for high-risk group; OR_L, odds ratio for low-risk group; Pperm, permutation p-value; MFS (Mean Factor Stability), average recurrence (%) of constituent factors across all reliable models; Score, weighted composite score = 0.7 × MFS + 0.3 × normalized Wmax (range 0–100). Priority classification: Highest (Score ≥ 80), High (60–79), Medium (40–59), Low (<40). Higher scores indicate greater model robustness and biological relevance.
