# Supplementary material for: From Serum to Genome: γ-Glutamyltransferase Gene Family Variants Shape Ischemic Stroke Risk via Sex-Specific Gene–Environment Interactions
Source: Life (Basel). 2026 Apr 24;16(5):721. doi: 10.3390/life16050721 (PMC13208725; doi:10.3390/life16050721)
Supplement: Supplementary file 1 [file life-16-00721-s001.zip › Supplementary Table S5.pdf]

Supplementary Table S5 eQTL-analysis of the studied polymorphisms

SNP, Assessed allele	Gene	Whole blood[1]		Arteries[2]		Brain[2]	
		P-value	Z-score	P-value	NES	P-value	NES
rs8140505, G	*GGT5*	-	-	-	-	-	-
	UPB1	$2.5x10^{-120}$	↓	-	-	-	-
	GSTT1	$4.3x10^{-63}$	↑	-	-	-	-
	SUSD2	$3.2x10^{-21}$	↑	-	-	-	-
	DDT	$1.4x10^{-16}$	↑	-	-	-	-
	POM121L9P	-	-	<0.0001	↓tibial	-	-
rs2275984, C	*GGT5*	$3.3x10^{-7}$	↓	0.001	↓aorta	<0.01	↓multiple
	UPB1	$8.9x10^{-149}$	↓	-	-	-	-
	GSTT1	$1.3x10^{-17}$	↓	-	-	-	-
	GGT1	0.000002	↓	-	-	-	-
	DDT	$1.2x10^{-11}$	↓	-	-	-	-
	SUSD2	$1.1x10^{-10}$	↓	-	-	-	-
	ADORA2A	$5.2x10^{-12}$	↑	-	-	-	-
	ADORA2A-AS1	-	-	0.0001	↑ tibial	-	-
	SPECC1L	$1.0x10^{-8}$	↓	0.0001	↓tibial	-	-
	POM121L9P	-	-	$5.4x10^{-17}$<br>$2.8x10^{-7}$<br>$9.2x10^{-15}$	↓aorta<br>↓coronary<br>↓tibial	<0.0001	↓multiple
rs2267073, T	*GGT5*	-	-	-	-	<0.01	↓multiple
	UPB1	$7.6x10^{-234}$	↑	$8.8x10^{-7}$<br>0.000002	↓aorta ↓tibial	-	-
	GSTT1	$5.6x10^{-57}$	↓	-	-	-	-
	ADORA2A	$9.1x10^{-20}$	↓	-	-	-	-
	ADORA2A-S1	-	-	0.000005	↑ tibial	-	-
	SUSD2	$3.9x10^{-18}$	↓	-	-	-	-
	DDT	$1.5x10^{-7}$	↓	-	-	-	-
	POM121L9P	-	-	$6.3x10^{-51}$<br>$2.5x10^{-31}$<br>$1.2x10^{-17}$	↓tibial ↓aorta<br>↓coronary	<0.00001	↓multiple
	GGTLC4P	-	-	0.00003	↓coronary		
rs11657054, G	*GGT6*	-	-	-	-	-	-
	MYBBP1A	$2.1x10^{-43}$	↓	$9.5x10^{-24}$<br>$3.3x10^{-14}$<br>$8.6x10^{-9}$	↑ tibial<br>↑ aorta<br>↑ coronary	<0.0001	↑hypothalamu<br>s
	CXCL16	$3.1x10^{-8}$	↑				
	ZNF232	-	-	-	-	0.00002	↓nucleus<br>accumbens
rs2100986, C	*GGT6*	-	-	-	-	-	-
	MYBBP1A	$9.3x10^{-43}$	↓	$3.4x10^{-21}$<br>$1.5x10^{-15}$<br>$6.2x10^{-10}$	↑ tibial<br>↑ aorta<br>↑ coronary	0.00003	↑hypothalamu<br>s
	CXCL16	$3.2x10^{-8}$	↑	-	-	-	-
	ALOX15	0.000001	↑	-	-	-	-
	PLD2	0.000008	↓	-	-	-	-
	SMTNL2	-	-	0.00013	↓tibial		

[1] Data of eQTLGen Consortium (www.eqtlgen.org); [2] Data of The Genotype-Tissue Expression (GTEx) Portal (https://gtexportal.org)
NES, Normalized Effect Size; Multiple depicts decreased gene expression in two or more regions of the brain.
